# Non-invasive neuroregulation techniques applied to patients with consciousness disorders: a narrative review

**DOI:** 10.3389/fnhum.2026.1790010

**Published:** 2026-05-08

**Authors:** Zhijie Cao, Hongzhen Du, Yufeng Zhou, Shutong Mei, Zhangyong Xia, Yang Wang

**Affiliations:** 1 First Clinical Medical College, Shandong University of Traditional Chinese Medicine, Jinan, Shandong, China; 2 The First School of Clinical Medicine of Binzhou Medical University, Binzhou Medical University Hospital, Binzhou, Shandong, China; 3 School of Special Education and Rehabilitation, Binzhou Medical University, Yantai, Shandong, China; 4 Department of Neurology, The Second People's Hospital of Liaocheng, Liaocheng, Shandong, China; 5 Department of Neurology, Liaocheng People's Hospital, Liaocheng, Shandong, China; 6 State Key Laboratory of Dampness Syndrome of Chinese Medicine, Shandong Subcenter, Liaocheng, Shandong, China

**Keywords:** disorders of consciousness, median nerve stimulation, neuromodulation, transcranial electrical stimulation, transcranial magnetic stimulation, vagus nerve stimulation

## Abstract

**Background:**

Disorders of consciousness (DoC) following severe brain injury pose significant diagnostic and therapeutic challenges. This narrative review comprehensively examines the current evidence for neuromodulation techniques in promoting consciousness recovery, including their mechanisms, efficacy, and future directions.

**Methods:**

We conducted a narrative review of evidence from randomized controlled trials, meta-analyses, and pilot studies investigating non-invasive and minimally invasive neuromodulation techniques for DoC. PubMed, Web of Science, Embase, CNKI, and Wanfang databases were searched from inception through December 2025. Techniques reviewed include transcranial magnetic stimulation (TMS), transcranial electrical stimulation (tES), median nerve stimulation (MNS), transcutaneous auricular vagus nerve stimulation (taVNS), transcranial focused ultrasound (tFUS), acupuncture, and spinal cord stimulation (SCS).

**Results:**

High-frequency repetitive TMS (10–20 Hz) targeting the dorsolateral prefrontal cortex showed significant CRS-R improvements with sustained effects. Right MNS (8 h/day, 2 weeks) significantly increased consciousness recovery rates in acute traumatic brain injury (RR = 1.36, 95% CI 1.18–1.56). Anodal tDCS demonstrated modest benefits, particularly in MCS patients. taVNS improved consciousness in MCS but not UWS patients. Short-term SCS (70 Hz) produced sustained improvements, especially in MCS − patients. Preliminary tFUS studies targeting the thalamus showed promising results. Combined interventions exhibited synergistic effects. Treatment responses varied by consciousness level, etiology, and individual factors. Adverse events were minimal across modalities.

**Conclusion:**

Emerging evidence suggests that neuromodulation may have therapeutic potential for DoC, with comparatively stronger support currently available for high-frequency rTMS, right MNS, and SCS. Future research should prioritize large-scale RCTs, predictive biomarker identification, personalized closed-loop protocols, and optimal combination strategies, with a gradual shift from deterministic to individualized treatment paradigmss.

## 1 Background

Disorders of Consciousness (DoC) are a group of clinical syndromes characterized by impaired or fluctuating wakefulness and awareness, which can be categorized into different levels based on behavioral manifestations (Teasdale and Jennett, 1974). Coma is defined by a persistent absence of the sleep–wake cycle, with patients keeping their eyes closed and showing no response to external stimuli (Comanducci et al., 2020). Patients in a Vegetative State (VS) (Laureys, 2005) or Unresponsive Wakefulness Syndrome (UWS) (Kondziella et al., 2020) may open their eyes but exhibit no signs of self- or environmental awareness. “Minimal Conscious State” (MCS) indicates the initial recovery of consciousness. It can be further divided into MCS − (which has non-verbal behaviors such as visual tracking or the ability to locate harmful stimuli) and MCS + (which has verbal behaviors such as command responses or clear verbal expressions) (Giacino et al., 2002). Emergence from MCS (eMCS) is defined by the ASPEN workgroup as the ability to functionally use objects or to engage in functional interactive communication, both of which must be demonstrated on consecutive evaluations (Thibaut et al., 2020). The fluctuations in the state of consciousness can last from several minutes to several days, which complicates the diagnosis and reduces its accuracy. The main causes of diffuse brain disease include traumatic brain injury, hypoxic–ischemic encephalopathy that occurs after cardiac arrest, cerebrovascular events (such as hemorrhagic stroke), and intracranial infections (Farisco et al., 2026). A 2022 public opinion survey jointly conducted by the United Kingdom and the United States estimated that the annual incidence rate of coma was 135 per 100,000 and 258 per 100,000, and the point prevalence rates of the patients were 7 per 100,000 and 31 per 100,000, respectively, (Bodien et al., 2025a). However, due to the lack of a structured monitoring system, unified definitions, and standardized diagnostic assessment tools in routine medical treatment, the epidemiological characteristics of DoC have still not been fully elucidated.

The DoC has imposed a heavy burden on the medical system and society. In intensive care settings, approximately 15 to 25% of patients who show no response actually exhibit cognitive motor dissociation (CMD), which means there is an extremely high risk of misdiagnosis (Bodien et al., 2024). Studies show that 30–80% of life-sustaining treatment withdrawal decisions are made within 72 h after the acute event. Given that a considerable proportion of patients may demonstrate neurological recovery beyond the acute phase, recent guidelines recommend postponing definitive poor-outcome prognostication until at least 28 days and adopting time-limited trials and phased communication to address early prognostic uncertainty and potential ethical risks (Williams et al., 2025). Long-term follow-up studies reveal that recovery of consciousness and function may continue for several years in some survivors (Magliacano et al., 2023); however, many patients will require institutional or home-based long-term care (Lissak and Young, 2024). Therapeutic strategies for DoC can be broadly divided into pharmacological and non-pharmacological interventions. In pharmacotherapy, stimulant-based interventions have been tested in the ICU acute phase. A randomized controlled trial demonstrated that in patients with acute DoC due to brain injury, a single dose of apomorphine or methylphenidate did not significantly improve pupillometric biomarkers of consciousness but transiently induced clinical arousal in approximately 20% of individuals (Othman et al., 2025). Other drugs under investigation include apomorphine, methylphenidate, s-citalopram, and emerging psychedelic compounds (Gao et al., 2023), however, clinical responses vary greatly and require further validation (Barra et al., 2024). Non-pharmacological treatments center on neuromodulation techniques. In addition, mesenchymal stem cell transplantation, as a neuroregenerative strategy, is in early clinical trial stages, while early multidisciplinary rehabilitation is thought to promote neuroplasticity (Durand et al., 2024), though the optimal timing and intensity of such interventions in ICU settings remain to be clarified.

Non-invasive Brain Stimulation (NIBS) refers to techniques such as Transcranial Magnetic Stimulation (TMS) and Transcranial Direct Current Stimulation (tDCS), which modulate cortical excitability and neuroplasticity without penetrating the skull. These approaches offer advantages of non-invasiveness, reversibility, favorable safety, and compatibility with behavioral tasks or neuroimaging, and have been widely applied in cognitive neuroscience and clinical treatment of neuropsychiatric disorders. However, current NIBS research faces challenges including limited reproducibility and marked interindividual variability in responsiveness, and the underlying mechanisms remain incompletely understood (Dong et al., 2023). This review aims to comprehensively and critically summarize the physical principles, stimulation parameters, evidence levels, and neural mechanisms of various non-invasive and minimally invasive neuromodulation techniques-including acupuncture-applied in DoC patients. It further provides a critical analysis of methodological limitations and, based on new perspectives of neural variability and brain-state dependency, proposes a future research roadmap transitioning from deterministic frameworks to probabilistic and individualized paradigms.

Beyond behavioral diagnosis, the neuroanatomical substrate of severe brain injury is also relevant to treatment response. Large-scale morphometric studies have demonstrated that cortical thickness and cortical surface area are genetically independent measures, each under the influence of distinct biological processes and developmental trajectories (Yu et al., 2023; Kuang et al., 2023). In a cohort of over 11,000 preadolescents. Identified both common and disorder-specific cortical thickness alterations across diagnostic families, with distinct genetic underpinnings: cortical thickness in transdiagnostically affected regions was associated with glutamatergic neurons, while disorder-specific regional thickness was linked to astrocytes, oligodendrocyte progenitor cells, and GABAergic neurons (Yu et al., 2023). Complementing these findings, Kuang et al. demonstrated that cortical surface area reduction was more pronounced in comorbid conditions and correlated with the general psychopathology factor across frontotemporal regions, whereas cortical thickness differences were associated with immune-related biological processes involving astrocytes and oligodendrocytes (Kuang et al., 2023). Although these studies were conducted in developmental psychiatric populations rather than in patients with acquired brain injury, they provide a conceptual framework for understanding how baseline cortical structural features and morphometric network organization may fundamentally shape cognitive and behavioral outcomes. In the context of DoC following severe brain injury, analogous structural alterations—including cortical thinning, gray matter loss, and disruption of large-scale morphometric network architecture—may similarly determine residual neural capacity and the potential to respond to neuromodulatory interventions.

### 1.1 Search strategy

This narrative review was conducted through a comprehensive literature search of PubMed, Web of Science, Embase, CNKI, and Wanfang databases from inception through December 2025. Search terms included combinations of “disorders of consciousness” OR “vegetative state” OR “unresponsive wakefulness syndrome” OR “minimally conscious state” OR “coma” AND each specific modality (e.g., “transcranial magnetic stimulation,” “transcranial direct current stimulation,” “median nerve stimulation,” “vagus nerve stimulation,” “focused ultrasound,” “acupuncture,” “photobiomodulation,” “hyperbaric oxygen,” “spinal cord stimulation”). Inclusion criteria comprised clinical studies (randomized controlled trials, quasi-RCTs, cohort studies, case series) and systematic reviews/meta-analyses involving adult patients with DoC receiving neuromodulation interventions, published in English or Chinese. Exclusion criteria included animal-only studies, conference abstracts without full-text publications, duplicate publications, and studies not reporting clinical or neurophysiological outcomes. Reference lists of included articles and relevant reviews were manually screened for additional eligible studies. As this is a narrative rather than systematic review, no formal PRISMA flow diagram, risk-of-bias assessment, or quantitative meta-analysis was performed.

### 1.2 Evidence grading

To ensure transparency and reproducibility, the overall strength of evidence for each neuromodulation modality was classified according to a modified Oxford Centre for Evidence-Based Medicine (OCEBM) framework adapted for the DoC field. Five evidence levels were applied: Level 1b (individual RCT or meta-analysis with consistent results from ≥2 RCTs); Level 2a (systematic review of cohort studies or meta-analysis of lower-quality RCTs); Level 2b (individual cohort study or lower-quality RCT); Level 3b (individual case–control or non-comparative cohort study); and Level 4 (case series, case reports, or expert opinion). Classification was performed independently by 2 authors, with disagreements resolved by discussion and consensus. The rationale for each modality’s assigned level, including key caveats, is summarized in Table 1. Evidence from different study designs is discussed with explicit annotation of study quality level throughout the text.

**Table 1:** Overall evidence strength for non-invasive neuromodulation modalities in disorders of consciousness—modified OCEBM framework.

Modality	Best available study design	No. of studies (DoC-specific)	Key evidence summary	Evidence level (modified OCEBM)	Rationale/Key caveats
High-frequency rTMS (10–20 Hz)	RCTs; meta-analysis	≥15 RCTs + 1 meta-analysis	Meta-analysis of 9 sham-controlled RCTs confirmed significant DLPFC rTMS benefit for pDoC (Gong et al., 2025). 10 Hz DLPFC rTMS (4 wks) significantly improved CRS-R with enhanced TMS-EEG connectivity persisting to week 8 (Liu et al., 2025); 20 Hz DLPFC rTMS improved CRS-R in a subset (Fan et al., 2022). Parietal 10 Hz rTMS improved CRS-R vs. sham (Wan et al., 2024a), increased alpha power (Xu et al., 2023), and modulated EEG microstate dynamics (Li Y. et al., 2024). 20 Hz M1 rTMS showed superior consciousness recovery vs. DLPFC or sham in post-traumatic VS (Shen et al., 2023). Angular gyrus (20 Hz) improved MCS but not UWS (Legostaeva et al., 2019). Multi-center personalized RCT protocol comparing DLPFC vs. parietal targets (Vitello et al., 2023). Additional sham-controlled studies with divergent findings at (Ge et al., 2021) group level (Cincotta et al., 2015; He et al., 2018; Zhang X. H. et al., 2021; Xia et al., 2017; He et al., 2020; Liu et al., 2018). MCS > UWS differential response consistently established.	1b	Largest body of sham-controlled RCT evidence in DoC field; meta-analysis (Gong et al., 2025) provides pooled confirmation. Limited by small per-study n (typically 10–40), heterogeneous targets (DLPFC, M1, parietal, angular gyrus), variable session numbers, and no adequately powered multicenter RCT yet completed. Biomarker-guided personalization (Vitello et al., 2023; He et al., 2020) may resolve inter-individual variability.
iTBS / Theta Burst Stimulation (TBS)	Randomized crossover studies	2	Single-session iTBS over left DLPFC did not improve CRS-R scores in VS patients but produced significant EEG spectral power, complexity, and interhemispheric frontal connectivity changes (Huang et al., 2024). Cerebellar TBS (CARE-DoC randomized crossover trial) was correlated with better functional outcomes and consciousness recovery at 6 months (Chen et al., 2025).	2b	Only two small randomized studies; protocols differ substantially (DLPFC vs. cerebellum; single vs. multi-session). No multicenter sham-controlled RCT conducted. Single-session designs (Huang et al., 2024) limit assessment of cumulative neuroplasticity; cerebellar findings (Chen et al., 2025) require replication.
Right Median Nerve Stimulation (RMNS)	Multicenter RCT + meta-analyses + earlier controlled trials	1 multicenter RCT + 2 meta-analyses (covering 18–23 studies, 1,831–1,856 patients) + 2 early RCTs	Large multicenter RCT (Wu et al., 2023) acute TBI coma patients (7–14 days post-injury) receiving 8 h/day RMNS × 2 weeks showed significantly higher 6-month consciousness recovery rate vs. controls, with faster improvements in GCS, FOUR, CRS-R, and GOSE (Wu et al., 2023). Meta-analysis (Yang et al., 18 RCTs, *N =* 1,831): consciousness recovery RR = 1.36 (95% CI 1.18–1.56, *p* < 0.001) at treatment end and RR = 1.31 at 6-month follow-up (Yang et al., 2025). Meta-analysis (Wang et al., 2022, 23 studies, *N =* 1,856): improved GCS, EEG grading, cerebral blood flow; dose–response relationship confirmed (Wang et al., 2022). Earlier controlled trials established foundational feasibility (Cooper et al., 1999; Peri et al., 2001).	1b	Highest-quality evidence in the DoC field; multicenter RCT (Wu et al., 2023) with adequate power; two independent meta-analyses (Yang et al., 2025; Wang et al., 2022) yielding consistent estimates. Primary limitation: most evidence is in acute traumatic coma; generalizability to non-traumatic etiology and chronic DoC requires dedicated studies.
Acupuncture	Meta-analysis of RCTs + additional systematic reviews	≥17 RCTs (*N =* 1,208, Huang et al., 2022) + additional SR	Huang et al. (2022) meta-analysis (17 RCTs, *N =* 1,208 stroke DoC patients): GCS improvement MD = 1.45 (95% CI 0.94–1.97, *p* < 0.0001); acupuncture+drugs vs. drugs alone RR = 1.68 (95% CI 1.30–2.18) for resuscitation rate; synergistic effects with rehabilitation/rTMS. Wu et al. (2024) systematic review confirmed beneficial effects of acupuncture for post-stroke coma (Wu et al., 2024). Most frequent acupoints: GV26 (Shuigou), PC6, GV20, SP6 (Guo et al., 2022).	2a	Evidence based on meta-analysis of multiple RCTs, but included trials carry high risk of bias (inadequate blinding, allocation concealment, outcome assessment). Predominantly China-based studies raise publication and regional bias concerns. Sham-acupuncture controls are rarely employed. Results most applicable to stroke-related DoC; trauma and anoxic etiologies underrepresented.
Spinal Cord Stimulation (SCS / st-SCS)	Pilot RCTs + prospective cohorts + TMS-EEG mechanistic studies	≥6 clinical studies	st-SCS (31 chronic pDoC patients, 2 weeks): significant CRS-R increase; MCS − showed greatest improvement; 70 Hz > 5 Hz with effects persisting 1 week post-treatment (Zhuang et al., 2022; Zhuang et al., 2024). In primary brainstem hemorrhage DoC (*n =* 14): >70% achieved ≥2-point CRS-R increase; 50% diagnostic category improvement (Huang et al., 2023b). EEG-based ‘ABCD’ model predicts SCS responders preoperatively (Sun et al., 2024). TMS-EEG after single SCS session: increased cortical complexity (PCI changes), especially in MCS; 50% MCS and 20% UWS showed more conscious behaviors at 6-month follow-up (Wang et al., 2023). Long-term outcome analysis (6 months): SCS feasible for promoting arousal; MCS and traumatic etiology associated with better prognosis (Yang et al., 2022).	2b	Evidence mainly from pilot and prospective studies with small-to-moderate sample sizes; no multicenter sham-controlled RCT yet published for st-SCS. Procedure is minimally invasive (cervical epidural placement) rather than fully non-invasive. EEG biomarker models (Sun et al., 2024) and TMS-EEG data (Wang et al., 2023) provide mechanistic support. Optimal frequency and duration remain to be standardized.
Anodal tDCS (DLPFC / M1)	Multicenter sham-controlled RCT + systematic review + multiple single-center RCTs and pilot studies	≥15 clinical studies	Systematic review confirmed DLPFC tDCS as most studied tES paradigm in pDoC (Liu et al., 2022). Triple-blind multicenter RCT (Thibaut et al., left DLPFC, CRS-R primary endpoint): no overall short-term superiority vs. sham; significant CRS-R improvements in MCS and traumatic subgroups at 3-month follow-up; no adverse effects (Thibaut et al., 2023). Bilateral M1 anodal tDCS in MCS: increased cortical oxygenation (fNIRS); reduced Ang-2, VEGF-C, IP-10 biomarkers (Straudi et al., 2023). Sequential bilateral DLPFC tDCS: N200/P300 latency reduction ~18/22 ms; EEG *β*-power +25%; CRS-R improved across arousal, visual, motor subscales (Gangemi et al., 2025). tDCS + robotic verticalization: enhanced P300 in MCS (Gangemi et al., 2024). Home-based 4-week tDCS (20 sessions) in chronic MCS: moderate improvement; safe (Martens et al., 2018). Network-based frontoparietal tDCS RCT: behavioral and EEG improvements (Martens et al., 2020). Single-session and HD-tDCS pilot studies (Hermann et al., 2020; Carrière et al., 2020; Thibaut et al., 2018; Wang et al., 2020; Guo et al., 2019). tDCS combined with median nerve stimulation: RCT protocol initiated (Chen et al., 2025).	2b	Primary multicenter RCT (Thibaut et al., 2023) yielded negative primary endpoint; subgroup and follow-up findings are hypothesis-generating. Large inter-individual variability and limited ability to personalize montage are key constraints. Systematic review (Liu et al., 2022) notes heterogeneous protocols across studies. HD-tDCS (Wang et al., 2020; Guo et al., 2019) may improve spatial focality; combination strategies (Martens et al., 2018; Martens et al., 2020) remain exploratory.
taVNS (Transcutaneous Auricular VNS)	RCTs + prospective cohort + longitudinal case studies + ongoing RCT protocols	3–5 RCTs/cohorts + 3 single-case/pilot reports	Randomized double-blind trial (Zhou et al., 2023): 4-week taVNS improved consciousness level in MCS but not UWS patients (Zhou et al., 2023). HRV-based taVNS response predicts short-term consciousness changes in DoC (Li et al., 2025). taVNS enhanced brain connectivity (EEG: *δ*↓ β↑) in MCS patients (Yifei et al., 2022). Long-term taVNS enhanced frontoparietal functional connectivity (fNIRS) and CRS-R in UWS (Gao F. et al., 2025). Six-month longitudinal case: CRS-R improved 4–6 to 12–13 (MCS/MCS+), *α*-peak emerged in EEG, HRV-HF increased (Osińska et al., 2022). Earlier feasibility studies confirmed MCS responded better than UWS (Noé et al., 2020). First taVNS case report monitored with fMRI (Yu et al., 2017). Cerebral hemodynamic changes monitored with fNIRS (Yu et al., 2021). Ongoing RCT protocols for long-term effects and taVNS+HD-tDCS combination (Vitello et al., 2023; Zhuang et al., 2023).	2b	Promising RCT data for MCS (Zhou et al., 2023) but no large multicenter trial completed. Protocol heterogeneity (frequency 20–25 Hz, pulse width 100–500 μs, intensity 0.1–8 mA) across studies limits comparability. UWS patients show limited behavioral response. Ongoing multicenter trials (Vitello et al., 2023; Zhuang et al., 2023) will clarify effect size and optimal parameters.
HBOT (Hyperbaric Oxygen Therapy)	Retrospective cohorts	2 large retrospective cohort studies	Li et al. retrospective cohort (484 DoC patients, 0.2 MPa, 2 ATA): total effective rate 67.1%; significantly improved GCS and Nanjing PVS Scale; early intervention (≤7 days), more sessions, higher baseline GCS, and traumatic etiology associated with better outcomes (Li S. et al., 2024). Zhao et al. (2025) pediatric cohort (255 children with DoC): confirmed importance of early HBOT and baseline neurological status in predicting consciousness recovery. Synergistic effects observed when combined with RMNS simultaneously in DoC (Liu et al., 2022).	3b	No sham-controlled RCT published for DoC; retrospective design limits causal inference and introduces selection bias. Both major studies are from Chinese single/few-center cohorts (Li S. et al., 2024; Zhao et al., 2025). Early intervention window and dose–response relationships are suggested but require prospective RCT validation. Widely used as adjunctive therapy in China.
tFUS (Transcranial Focused Ultrasound)	Proof-of-concept / single-arm pilot studies	3 small studies (*N =* 1–11 patients each)	First human case report (Monti et al., 2016): thalamic tFUS (650 kHz, PRF 100 Hz) in acute DoC; CRS-R improved 14–15 → 13–17; regained language comprehension within 3 days; attempted walking by day 5 (Monti et al., 2016). Cain et al. (2022) (11 acute DoC): single session thalamic tFUS significantly increased CRS-R; 4 patients showed diagnostic category improvement (Cain et al., 2022). Cain et al. (2022) (10 chronic DoC): 2 sessions 1 week apart; 4 patients improved; CRS-R showed significant linear increase (Cain et al., 2021). No serious adverse events across 22 patients in 3 studies.	3b	Early-phase proof-of-concept evidence; extremely small samples (*n =* 1–11) and no sham controls. Optimal parameters (frequency, PRF, intensity, target) remain unknown. Deep target access (thalamus) is a key advantage over TMS/tDCS. Rapid translation from preclinical to clinical remains a distinguishing feature; adequately powered sham-controlled RCTs urgently needed.
Light Therapy / Circadian Regulation	Small pilot controlled studies	2 studies (*N =* 8–17 patients each)	Angerer et al. (2022) DoC patients, 7–8 days): simulated dynamic daylight vs. standard ward lighting restored body temperature cycles close to 24 h in 11 patients, with increased rhythm strength and daytime stability. Yelden et al. (2022) DoC patients, 3 × 1 h/day blue-light-enriched glasses × 1 week): 2 patients improved diagnostically from UWS to MCS; sleep–wake pattern improvements in combined 17-patient melatonin + blue-light study over 5 weeks (Yelden et al., 2022).	3b	Small samples and uncontrolled or minimally controlled designs. Endpoints are indirect (circadian stability, sleep–wake patterns) rather than established consciousness measures (CRS-R). Effects difficult to disentangle from co-interventions. Mechanistically plausible via suprachiasmatic nucleus and melatonin pathways (Gooley et al., 2011; Lucas et al., 2014). Larger sham-controlled studies warranted.
tACS (Gamma / Alpha)	Pilot / single-session studies	1–2 small DoC studies	Naro et al. (2016): gamma-band tACS significantly enhanced cortical effective connectivity in MCS and some UWS patients; first demonstration of tACS as potential diagnostic stratification tool in DoC. Bartolini et al. (2025) functional connectivity stability in the alpha band markedly reduced in DoC and positively correlated with consciousness level; provides theoretical basis for alpha-band tACS targeting (Bartolini et al., 2025).	4	Only one direct DoC interventional study with tACS (Naro et al., 2016); the second study (Bartolini et al., 2025) is a neuroimaging/connectivity study providing indirect evidence. Mechanism (neural entrainment) not experimentally validated in DoC populations. Small samples, no sham control, and single-session designs; optimal frequency (gamma vs. alpha) and montage undetermined.
tPBM (Photobiomodulation)	Case reports + preclinical studies	1–2 case reports in severe TBI; supporting animal model data	Naeser et al. (2011) case reports (2 chronic severe TBI patients, 810 nm NIR): improved cerebral blood flow velocity and neuropsychological functions after multi-session tPBM. Preclinical evidence: a single transcranial irradiation with red light (660 nm) or near-infrared light (810 nm) 4 h post-TBI improved neurological severity scores; 3 consecutive days more effective than single dose, reducing neuronal degeneration and promoting neurogenesis/BDNF (Montazeri et al., 2021). Direct DoC clinical evidence remains extremely limited.	4	Extremely limited clinical evidence specifically in DoC patients; primary evidence comes from TBI case reports (Naeser et al., 2011) and animal models (Montazeri et al., 2021). Significant skull attenuation of light limits photon delivery to deep brain structures relevant to consciousness (thalamus, brainstem). Optimal parameters highly variable across studies (wavelength 630–1,100 nm, power density 0.01–50 mW/cm^2^). Safety profile is favorable; awaits controlled clinical investigation.
tRNS (Random Noise Stimulation)	Single pilot study	1 (*n =* 9 VS-UWS patients)	Mancuso et al. (2017) VS-UWS patients, bilateral DLPFC tRNS, multiple sessions): no significant improvement in consciousness level or EEG measures vs. sham; only 1 patient transitioned from VS-UWS to MCS after stimulation (Mancuso et al., 2017).	2b	Single underpowered study (*n =* 9) with no replication (Mancuso et al., 2017). Primary outcome was negative. Mechanistic rationale (stochastic resonance) is theoretically plausible but not validated in DoC. Requires independent replication before further clinical investigation can be prioritized.

Evidence Level Key (Modified OCEBM Framework): 1b = Individual RCT or meta-analysis with consistent results from multiple RCTs (high-quality evidence, strong recommendation). 2a = Systematic review of cohort studies or meta-analysis of lower-quality RCTs (moderate-high evidence). 2b = Individual cohort study or lower-quality RCT (moderate evidence). 3b = Individual case–control study or non-comparative cohort (limited evidence). 4 = Case series, case reports, or expert opinion (very limited / preliminary evidence). Evidence levels follow a modified Oxford Centre for Evidence-Based Medicine (OCEBM) framework adapted for the DoC neuromodulation field. Reference numbers in square brackets correspond to the numbered reference list in the main manuscript. DoC, disorder of consciousness; MCS, minimally conscious state; UWS, unresponsive wakefulness syndrome; VS, vegetative state; TBI, traumatic brain injury; pDoC, prolonged DoC; CRS-R, Coma Recovery Scale-Revised; GCS, Glasgow Coma Scale; RCT, randomized controlled trial; SR, systematic review; CI, confidence interval; RR, risk ratio; MD, mean difference; DLPFC, dorsolateral prefrontal cortex; M1, primary motor cortex; EEG, electroencephalography; fNIRS, functional near-infrared spectroscopy; fMRI, functional MRI; PCI, Perturbational Complexity Index; HRV, heart rate variability.

### 1.3 TMS

TMS is a non-invasive neuromodulation technique based on the principle of electromagnetic induction. Its core mechanism involves generating a rapidly changing magnetic field through a specific coil placed over the scalp. This magnetic field penetrates the skull and induces an electric current in cortical tissue, thereby influencing neuronal depolarization and modulating cortical excitability (Lin et al., 2025; Huang et al., 2023a). In terms of stimulation frequency, low-frequency stimulation (≤1 Hz) generally produces an inhibitory effect, reducing neuronal excitability, local metabolic levels, and cerebral blood flow, whereas high-frequency stimulation (5–20 Hz) enhances cortical excitability and exerts more sustained after-effects. Coil types primarily include figure-eight coils and deep stimulation coils-the former providing higher spatial focality, while the latter can reach deeper brain structures (Bhattacharya et al., 2025). Stimulation intensity is usually determined relative to the resting motor threshold (Schuler et al., 2025). rTMS is currently the most widely applied TMS modality in the treatment of DoC. It achieves sustained modulation of cortical function through sequences of multiple pulses delivered at specific frequencies. In recent clinical studies, high-frequency rTMS (10–20 Hz) has accumulated relatively robust evidence in patients with pDoC (Gong et al., 2025). Four weeks of 10 Hz rTMS applied to the left DLPFC significantly enhanced TMS-evoked connectivity assessed by TMS-EEG, accompanied by a notable increase in CRS-R scores, with the advantage persisting to week 8 (Liu et al., 2025). Fan et al. (2022) administered 20 Hz rTMS over the left DLPFC in 40 DoC patients and found that CRS-R scores significantly improved in some patients after intervention, suggesting that 20 Hz rTMS may facilitate consciousness recovery in a subset of individuals. Additionally, applying 10 Hz rTMS to the parietal cortex for 10 consecutive days significantly improved CRS-R scores compared with sham stimulation (Wan et al., 2024a), and increased alpha power (Xu et al., 2023) and EEG microstate dynamics (Li Y. et al., 2024) in pDoC patients. It is noteworthy that heterogeneity in therapeutic efficacy exists across studies. He et al. (2018) and Liu et al. (2018) reported that five sessions of rTMS targeting the Primary Motor Cortex (M1) showed trends of enhanced functional connectivity on neuroimaging, but behavioral assessments did not reach statistical significance at the group level. This indicates that an adequate duration of stimulation may be crucial for clinical efficacy. There are reports suggesting that applying 20-hertz repetitive transcranial magnetic stimulation to the M1 brain area can significantly promote the recovery of consciousness in patients with post-traumatic, and its efficacy is superior to that of frontal lobe cortex stimulation or sham stimulation (Shen et al., 2023). Furthermore, Legostaeva et al. targeted the stimulation at the angular gyrus - a multimodal integration center closely connected to the default mode network - and found that after 10 sessions of 20-hertz stimulation treatment for MCS patients, the MCS indicators of the patients improved, while no significant changes were observed in the UWS group. A multicenter randomized and personalized controlled trial compared the DLPFC and parietal lobe repetitive transcranial magnetic stimulation therapies for DoC patients, and the results suggested that both stimulation protocols may improve consciousness and neurophysiological functions in some patients; however, the individual differences were substantial. These findings suggest that the optimal stimulation target for consciousness recovery may need to be individualized and may eventually be informed by electroencephalographic biomarkers (Vitello et al., 2023).

The application of Theta Burst Stimulation (TBS) in DoC remains at an exploratory stage. TBS includes two modes—intermittent TBS (iTBS) and continuous TBS (cTBS)—and is characterized by its shorter stimulation duration (Luo et al., 2025), typically requiring only a few minutes to induce cortical plasticity effects comparable to or greater than conventional rTMS (Li et al., 2026). A recent randomized crossover study in VS patients found that a single iTBS session applied to the left DLPFC did not significantly improve CRS-R scores but markedly altered EEG spectral power, complexity, and functional connectivity, particularly enhancing interhemispheric frontal connectivity (Huang et al., 2024). Another study targeting the cerebellum reported that active treatment was correlated with better functional outcomes and consciousness recovery at 6 months (Chen et al., 2025).

The selection of stimulation targets is one of the key determinants of TMS efficacy. The DLPFC, as a central hub of the frontoparietal network, plays a critical role in higher-order cognitive functions, executive control, and motor planning (Anderson et al., 2025), and is anatomically connected to multiple cortical regions (Avalos-Alais et al., 2026). The participation coefficient of the frontal network correlates positively with the level of consciousness (Block, 2024; Mashour et al., 2022), and MCS patients exhibit significantly higher alpha-band phase-phase coupling and clustering coefficients in the frontal cortex compared to UWS patients (Ma et al., 2024). Stimulating the DLPFC can influence widespread network activity, including posterior cortical regions, through remote modulation effects, providing neurophysiological support for its role as a preferred target (Chau et al., 2025). The M1 is also considered a potential target due to its tight coupling with the sensorimotor network and the feasibility of objectively assessing physiological effects via motor evoked potentials (Bertoni et al., 2025). The precuneus/posterior cingulate cortex, as a core node of the Default Mode Network, is closely associated with self-awareness and internal consciousness (Fransson and Marrelec, 2008; Crone et al., 2015). A pilot study of parietal repetitive TMS has provided preliminary evidence that targeting the parietal cortex may modulate awareness in DoC patients (Wan et al., 2024a), and the posterior transient state characterized by enhanced parietal alpha power and connectivity occurs more frequently in healthy individuals than in DoC patients (Zhang Y. et al., 2025). Some researchers have therefore proposed shifting stimulation targets from the frontal lobe, which is often affected by traumatic injury, to the posterior parietal regions.

The mechanisms underlying TMS-induced consciousness recovery likely operate on multiple levels. First, *in vitro* and animal studies suggest that rTMS can induce synaptic plasticity resembling Long-Term Potentiation (LTP) or Long-Term Depression (LTD) (Galanis et al., 2025). Second, TMS modulates the excitation-inhibition balance at both local and network levels, affecting GABAergic and glutamatergic neurotransmission (Barone et al., 2025). In human studies, He et al. observed elevated serum estradiol levels following rTMS treatment, and given that estradiol exerts regulatory effects on cortical excitability, this finding suggests that neuroendocrine mechanisms may contribute to the therapeutic effects of TMS (He et al., 2021). The TMS-EEG combined approach offers a valuable window into elucidating TMS mechanisms (Bai et al., 2024). Systematic reviews have identified the Perturbational Complexity Index (PCI) as an effective biomarker for distinguishing VS/UWS from MCS, quantifying the brain’s capacity for information integration by assessing the spatiotemporal complexity of TMS-evoked activity (Xu et al., 2024). VS/UWS patients display characteristically simplified neural response patterns and OFF-periods (Rosanova et al., 2018), with TMS-induced causal effects on local cortical activity lasting significantly shorter than in healthy awake controls. Moreover, human neurophysiological studies suggest that TMS can reshape large-scale brain networks and enhance fronto-posterior functional connectivity (He et al., 2025). In MCS patients, TMS evokes frontal-to-parietal responses, which are absent in VS/UWS patients—consistent with the critical role of frontoparietal network integrity in sustaining consciousness (Zhao et al., 2024; Ping et al., 2025). In recent years, there has been a growing number of studies exploring brain function in patients with DoC using TMS. Table 2 summarizes key information regarding patient populations, stimulation protocols, physiological assessments, and main findings from relevant studies.

**Table 2:** Summary of TMS studies in disorders of consciousness.

Study/Author	Sample	Target	Parameters	Duration	Main outcomes
Liu et al. (2025)	pDoC patients	Left DLPFC	10 Hz rTMS	4 weeks	Significant increase in CRS-R scores; enhanced TMS-evoked connectivity persisting to week 8
Fan et al. (2022)	40 DoC patients	Left DLPFC	20 Hz rTMS	Multiple sessions	CRS-R scores significantly improved in some patients
Wan et al. (2024a)	pDoC patients	Parietal cortex	10 Hz rTMS	10 days	Significant CRS-R improvement vs. sham
Xu et al. (2023)	pDoC patients	Posterior parietal cortex	10 Hz rTMS	Multiple sessions	Improved functional recovery; increased alpha power
Li et al. (2024)	MCS patients	Parietal cortex	10 Hz rTMS	Multiple sessions	Modulation of EEG microstate dynamics
Shen et al. (2023)	Post-traumatic VS	M1	20 Hz rTMS	Multiple sessions	Significant consciousness recovery; superior to DLPFC or sham
Legostaeva et al. (2019)	MCS and UWS patients	Angular gyrus	20 Hz rTMS	10 sessions	Improvement in MCS patients; no change in UWS
Huang et al. (2024)	VS patients	Left DLPFC	iTBS (single session)	Single session	No CRS-R improvement; altered EEG spectral power, complexity, and connectivity
Chen et al. (2025)	DoC patients	Cerebellum	TBS	Multiple sessions	Active treatment correlated with better outcomes at 6 months
Vitello et al. (2023)	90 subacute/chronic DoC patients (planned)	Left DLPFC and left angular gyrus	20 Hz rTMS, 120% RMT, 3200 pulses	Multiple sessions	Protocol for multicenter RCT comparing frontal vs. parietal rTMS; personalized treatment approach (not yet completed)
Cincotta et al. (2015)	11 VS/UWS patients	M1	20 Hz rTMS	5 sessions	No significant behavioral improvement at group level
He et al. (2018)	DoC patients	M1	20 Hz rTMS	5 sessions	Only 1 patient showed improvement; EEG changes observed
Zhang et al. (2021)	Persistent VS/UWS patients	Left DLPFC	10 Hz rTMS	Multiple sessions	About 25% showed consciousness improvement
Xia et al. (2017)	MCS and UWS patients	Left DLPFC	10 Hz rTMS	Multiple sessions	MCS showed more significant improvement than UWS
Ge et al. (2021)	VS patients	Right DLPFC	10 Hz rTMS	Multiple sessions	Shortened MEP latency; CNS conduction enhanced
He et al. (2020)	DoC patients	DLPFC	20 Hz rTMS	Multiple sessions	Higher baseline α power and lower δ power predicted response
Liu et al. (2018)	DoC patients	M1	20 Hz rTMS	5 sessions	Enhanced functional connectivity trend but no significant behavioral change

TMS, transcranial magnetic stimulation; rTMS, repetitive TMS; iTBS, intermittent theta burst stimulation; TBS, theta burst stimulation; DLPFC, dorsolateral prefrontal cortex; M1, primary motor cortex; pDoC, prolonged disorders of consciousness; DoC, disorders of consciousness; VS, vegetative state; UWS, unresponsive wakefulness syndrome; MCS, minimally conscious state; CRS-R, Coma Recovery Scale-Revised; EEG, electroencephalography; MEP, motor evoked potential; RMT, resting motor threshold; CNS, central nervous system; RCT, randomized controlled trial.

### 1.4 Transcranial electrical stimulation (tES)

tES refers to a class of non-invasive techniques that modulate brain activity by delivering low-intensity electrical currents, typically with a peak amplitude of 1–4 mA, to the scalp (Nejati et al., 2025). Unlike TMS, which directly induces neuronal action potentials, tES exerts subthreshold modulation of the neuronal membrane potential-depolarizing or hyperpolarizing it slightly—to alter neuronal firing thresholds and excitability without directly triggering action potentials (Sveva et al., 2025). One inherent limitation of tES lies in its relatively low spatial resolution, as the current tends to disperse diffusely between surface electrodes (Aberra et al., 2023). Finite element modeling based on individual MRI data can simulate electric field propagation across different brain tissues, thus guiding precise electrode placement to maximize electric field strength in the target region while minimizing stimulation of non-target areas (Ma et al., 2024).

#### 1.4.1 tDCS

tDCS delivers a constant direct current between electrodes to achieve neuromodulation, with effects that are polarity-dependent (Aloi et al., 2021). Anodal stimulation generally increases local cortical excitability, whereas cathodal stimulation produces inhibitory effects (Song et al., 2025). In addition, tDCS may act via activation of trigeminal nerve pathways projecting to the brainstem (Majdi et al., 2025). An interventional study applying bilateral M1 anodal tDCS in MCS patients demonstrated increased cortical oxygenation on fNIRS after a single session, along with decreases in serum Ang-2, VEGF-C, and IP-10 levels after 10 sessions. CRS-R scores correlated with TNFα and IP-10 (Straudi et al., 2023). In the field of DoC, anodal tDCS over the DLPFC is the most extensively investigated paradigm (Liu et al., 2022). A sequential bilateral DLPFC tDCS protocol showed that after intervention, N200 and P300 latencies were reduced by approximately 18 ms and 22 ms, respectively, while EEG *β*-band power increased by about 25%. CRS-R scores improved from baseline, with seven patients showing total score increases primarily in arousal, visual, and motor subscores (Gangemi et al., 2025). Another quasi-randomized study found that tDCS significantly enhanced P300 latency responses during robotic verticalization training in MCS patients (Gangemi et al., 2024). Clinical studies are also exploring the combination of tDCS with personalized music interventions (Spaccavento et al., 2024). In a triple-blind multicenter RCT, anodal tDCS was applied to the left DLPFC of DoC patients during the rehabilitation phase, with CRS-R as the primary endpoint. The short-term intervention group showed no superiority over sham stimulation and was terminated early due to insufficient power; however, subgroup analysis at three-month follow-up revealed statistically significant CRS-R improvements in MCS and traumatic etiology patients, with no adverse effects observed (Thibaut et al., 2023). Cathodal tDCS in DoC has been less studied. Its theoretical rationale lies in suppressing local hyperexcitability or pathological oscillatory activity, though systematic clinical evidence remains limited (Reale et al., 2025). High-definition tDCS (HD-tDCS) replaces traditional large pad electrodes with multiple small circular electrodes (<12 mm diameter). Using a ‘4 × 1’ montage-one central anode surrounded by four cathodes-this approach markedly improves spatial focality. Such refinement mitigates the coarse localization and current spread limitations of conventional tDCS. Early applications in pDoC suggest potential for more precise target modulation, though related clinical research remains preliminary (Huang et al., 2025). tDCS combined with passive thumb mobilization exhibits both short- and long-range effects on motor network dynamics during command-following tasks. It can modulate M1 self-inhibition and thalamocortical excitation toward M1 (Aloi et al., 2022), with anodal effects becoming more pronounced than cathodal ones after five stimulation sessions (Aloi et al., 2023). Randomized controlled trials on long-term tDCS application have also been conducted, comparing the effects of tDCS combined with median nerve stimulation, sham tDCS combined with median nerve stimulation, and tDCS combined with sham median nerve stimulation on CRS-R outcomes. The intervention lasted 4 weeks with a 24-week follow-up, and the primary endpoint was the change in total CRS-R score (Chen et al., 2025).

#### 1.4.2 Transcranial alternating current stimulation (tACS)

tACS delivers sinusoidal alternating currents that interact with endogenous brain oscillations through the mechanism of ‘neural entrainment’. When the exogenous oscillatory frequency approximates the endogenous rhythm, specific frequency-band activity can be tuned and enhanced (Krugliakova et al., 2025). tACS has shown clear effects on cognitive performance (Mitterová et al., 2025), particularly in improving memory (Booth et al., 2022). Clinical studies applying tACS in DoC are still limited. It has been demonstrated that tACS targeting the individual somatosensory alpha frequency over the sensorimotor cortex can modulate pain perception (Fathi et al., 2025) and improve visuospatial and executive functions in patients with Lewy body dementia in randomized controlled trials (Benussi et al., 2024). Naro et al. were the first to apply gamma-band tACS in DoC patients, finding that stimulation significantly enhanced cortical effective connectivity in MCS and some UWS patients, suggesting that tACS may help reveal residual consciousness and support diagnostic stratification (Naro et al., 2016). Bartolini et al. employed time-varying graph analysis and found that functional connectivity stability in the alpha band was markedly reduced in DoC patients and positively correlated with consciousness levels. These findings indicate that alpha rhythms play a key role in maintaining consciousness and provide a theoretical basis for future tACS studies targeting the alpha frequency band (Bartolini et al., 2025).

#### 1.4.3 Transcranial random noise stimulation (tRNS) and transcranial pulsed current stimulation

tRNS delivers alternating currents with randomly varying frequencies and amplitudes, and its mechanism may involve the stochastic resonance effect (Uner et al., 2025), in which random noise enhances the detectability of near-threshold signals and repeatedly opens voltage-gated sodium channels to promote cortical excitability (Wei et al., 2025). Mancuso et al. conducted a study on nine VS–UWS patients applying tRNS over bilateral DLPFC but found no significant improvement in consciousness level or EEG measures compared with sham stimulation, with only one patient transitioning from VS–UWS to MCS after stimulation (Mancuso et al., 2017). Applications of tRNS in the field of DoC therefore remain to be further explored. Research on tES (transcranial electrical stimulation) intervention for disorders of consciousness focuses on its therapeutic potential and neuromodulatory effects, with key information from relevant clinical studies summarized in Table 3.

**Table 3:** Summary of transcranial electrical stimulation (tES) studies in disorders of consciousness.

Study/Author	Sample	Target	Parameters	Duration	Main outcomes
Straudi et al. (2023)	MCS patients	Bilateral M1	Anodal tDCS	10 sessions	Increased cortical oxygenation (fNIRS); decreased Ang-2, VEGF-C, IP-10; CRS-R correlated with TNFα and IP-10
Gangemi et al. (2025)	MCS patients	Bilateral DLPFC	Sequential tDCS	Multiple sessions	N200/P300 latency reduced ~18/22 ms; increased EEGβ-band power; CRS-R improved
Gangemi et al. (2024)	MCS patients	DLPFC	tDCS + robotic verticalization	Multiple sessions	Enhanced P300 latency responses
Thibaut et al. (2023)	DoC patients (multicenter RCT)	Left DLPFC	Anodal tDCS	Short-term	No superiority over sham short-term; significant CRS-R improvement in MCS and traumatic subgroups at 3-month follow-up
Aloi et al. (2022)	Healthy/DoC	M1	tDCS + passive mobilization	Multiple sessions	Modulated M1 self-inhibition and thalamocortical excitation
Aloi et al. (2023)	Healthy subjects	M1	Multi-session tDCS + passive mobilization	5 sessions	Anodal effects more pronounced than cathodal after 5 sessions
Chen et al. (2025)	pDoC (cerebral hemorrhage)	DLPFC	tDCS + median nerve stimulation	4 weeks + 24-week follow-up	RCT protocol: comparing combined vs. single modality on CRS-R
Naro et al. (2016)	MCS and UWS	Frontal cortex	Gamma-band tACS	Single/multiple sessions	Enhanced cortical effective connectivity in MCS and some UWS patients
Mancuso et al. (2017)	9 VS-UWS patients	Bilateral DLPFC	tRNS	Multiple sessions	No significant improvement; only 1 patient transitioned to MCS
Zhang et al. (2021)	pDoC patients	Multiple targets	Multi-target tDCS	80 sessions	Both MCS and UWS showed significant improvement
Hermann et al. (2020)	DoC patients	Left DLPFC	Anodal tDCS	Single session	Combined behavioral and EEG evidence for direct cortical effect
Carrière et al. (2020)	pDoC patients	Left DLPFC	Anodal tDCS	Single session	θ-α power changes in responders; pilot double-blind RCT
Thibaut et al. (2018)	DoC patients	Left DLPFC	Anodal tDCS	Single session	Theta network centrality correlated with tDCS response
Wang et al. (2020)	DoC patients	Precuneus	HD-tDCS	14–21 sessions	Improved CRS-R and MMN; long-lasting effects
Guo et al. (2019)	Chronic DoC	Precuneus	HD-tDCS	Multiple sessions	δ power decreased, α power increased; CRS-R improved
Martens et al. (2018)	Chronic MCS	Left DLPFC	Home-based tDCS	4 weeks (20 sessions)	Moderate improvement; safe for home use
Martens et al. (2020)	Severe brain injury	Frontoparietal network	Network-based tDCS	Multiple sessions	Behavioral and EEG improvements in RCT

tES, transcranial electrical stimulation; tDCS, transcranial direct current stimulation; tACS, transcranial alternating current stimulation; tRNS, transcranial random noise stimulation; DLPFC, dorsolateral prefrontal cortex; M1, primary motor cortex; MCS, minimally conscious state; UWS, unresponsive wakefulness syndrome; VS, vegetative state; pDoC, prolonged disorders of consciousness; CRS-R, Coma Recovery Scale-Revised; RCT, randomized controlled trial; fNIRS, functional near-infrared spectroscopy; Ang-2, angiopoietin-2; VEGF-C, vascular endothelial growth factor C; IP-10, interferon gamma-induced protein 10; TNFα, tumor necrosis factor alpha; EEG, electroencephalography; HD-tDCS, high-definition transcranial direct current stimulation; MMN, mismatch negativity; N200, event-related potential N200 component; P300, event-related potential P300 component; θ, theta; α, alpha; β, beta; δ, delta.

### 1.5 Median nerve electrical stimulation (MNS/MNES)

MNS/MNES is a non-invasive neuromodulation technique that indirectly activates the central nervous system by stimulating peripheral nerves (Yang et al., 2025), sharing mechanistic parallels with other peripheral cranial nerve stimulation approaches that act via brainstem pathways. Its anatomical foundation lies in the disproportionately large cortical representation of the median nerve within the somatosensory cortex (Majdi et al., 2025; Merzenich and Jenkins, 1993). The cortical area representing the hand occupies a much greater region than its actual surface area, allowing median nerve stimulation to induce widespread cortical activation (Allison et al., 1991). The arousal-promoting mechanisms of MNS involve multiple neural pathways. First, electrical stimulation signals are transmitted via the median nerve to the dorsal horn of the spinal cord, ascending through the medial lemniscus to the cuneate nucleus (Wang et al., 2012), then projecting to the ventral posterolateral nucleus of the thalamus and ultimately activating the somatosensory cortex (Day et al., 2001; Tsai et al., 2007). Studies characterizing primary sensory neuron responses following median nerve injury have further confirmed the involvement of these afferent pathways. More importantly, this afferent pathway maintains close connections with the brainstem reticular formation—stimulation-induced sensory input may help disrupt the sleep-like cortical OFF-periods characteristic of UWS patients, thereby facilitating the restoration of arousal. (Rosanova et al., 2018; Tsai et al., 2007). Animal studies have further elucidated the molecular mechanisms underlying MNS. Feng et al. found that in animal experiments, MNS exerts arousal-promoting effects by modulating the expression of orexin-A and its receptor Orexin receptor 1 in the hypothalamus (Feng and Du, 2016). Moreover, functional neuroimaging studies have shown that MNS significantly increases blood flow and BOLD signals in the contralateral sensorimotor cortex, suggesting that it modulates hemodynamic responses associated with neuronal activity (Mullinger et al., 2013).

Alterations in neurotransmitter metabolism-particularly dopaminergic signaling-may also contribute to the effects of MNS. In a study involving five patients in coma due to severe TBI who underwent right-sided MNS for 2 weeks, high-throughput transcriptome sequencing of cerebrospinal fluid revealed that MNS significantly altered microRNA expression profiles. These miRNAs were mainly enriched in biological pathways related to neuronal growth, synaptic plasticity, signal transduction, and inflammation regulation. Therefore, in human patients, the currently available evidence supports transcriptomic changes associated with neuroprotection and neurotransmitter metabolism after MNS, whereas specific downstream effects such as inhibition of neuronal apoptosis or enhancement of dopaminergic signaling should still be regarded as mechanistic inferences rather than directly demonstrated clinical mechanisms (Jia et al., 2022).

A growing body of clinical evidence suggests potential efficacy of MNS, particularly in acute traumatic coma. A multicenter randomized controlled trial by Wu et al. provided evidence supporting right-sided MNS. The study demonstrated that acute comatose patients 7–14 days post-TBI who received 8 h per day of right MNS (RMNS) for 2 consecutive weeks showed a significantly higher rate of consciousness recovery at 6 months compared with controls, along with faster and greater improvements in multiple functional indices including GCS, FOUR, CRS-R, EEG scores, and GOSE scores (Wu et al., 2023). Yang et al. conducted a systematic review including 18 randomized controlled trials involving 1,831 TBI patients, and meta-analysis indicated that the proportion of consciousness recovery was significantly higher in the MNS group than in controls, both at the end of treatment (RR = 1.36, 95% CI 1.18–1.56, *p* < 0.001) and at 6-month follow-up (RR = 1.31, 95% CI 1.16–1.47, *p* < 0.001) (Yang et al., 2025). Similarly, a meta-analysis by Wang et al. including 23 studies with 1,856 patients further confirmed that MNS improved GCS scores, EEG grading, mean cerebral blood flow velocity, and systolic cerebral blood flow velocity, while reducing disability rating scale scores and ICU length of stay. Subgroup analyses revealed a dose–response relationship, indicating that longer treatment durations were associated with greater GCS improvements (Wang et al., 2022). Compared with single-modality interventions using either rTMS or MNS alone, combined stimulation—applied to the right wrist in pDoC patients—further enhanced CRS-R and GCS scores, improved SEP N20 amplitude and latency, and modulated EEG activity (Xiong et al., 2023). In addition, Liu et al. suggested that hyperbaric oxygen therapy and right-sided MNS may have synergistic effects. The combined intervention was associated with further improvement in GCS and neurophysiological measures (EEG, brainstem auditory evoked potentials [BAEP], and upper-limb somatosensory evoked potentials [USEP]), and the in-chamber simultaneous protocol appeared to show the greatest observed benefit (Liu et al., 2022).

### 1.6 Transcutaneous auricular vagus nerve stimulation (taVNS)

taVNS is a novel non-invasive neuromodulation technique that achieves indirect regulation of the central nervous system by stimulating the auricular branch of the vagus nerve distributed in the cymba conchae (Courties et al., 2025). The anatomical basis of this technology lies in the fact that the vagus nerve has a unique afferent branch reaching the body surface in the outer ear, making transcutaneous stimulation possible (Zou et al., 2024). The neural pathway of taVNS follows a “bottom-up” activation pattern (Hilz, 2022). The stimulation signals are first transmitted via the afferent fibers of the vagus nerve to the Nucleus Tractus Solitarius (NTS), and then projected to several key brainstem nuclei, including the Locus Coeruleus (LC) and the Dorsal Raphe Nucleus (DRN) (Ma L. et al., 2025). The LC is the main source of norepinephrine (NE) in the brain, while the DRN is the primary site of serotonin (5-HT) production. The activation of these nuclei is hypothesized to affect multiple brain regions, such as the prefrontal cortex, thalamus, hypothalamus, amygdala, and cingulate gyrus, thereby contributing to broader regulation of cortical brain activity (Tong et al., 2025; Chen et al., 2023). The mechanism by which taVNS promotes consciousness recovery likely involves multiple levels. First, activation of the LC-norepinephrine system has been proposed as a core mechanism, because norepinephrine is an important mediator of arousal (Tong et al., 2025), and increased norepinephrine signaling may support functional recovery after severe brain injury. Studies on the P300 event-related potential provide electrophysiological evidence supporting this hypothesis (Giraudier et al., 2024; Gurtubay et al., 2023). Second, in animal experiments, taVNS has been reported to enhance cerebral blood flow and increase blood oxygen level-dependent signals and functional connectivity in regions such as the prefrontal cortex, thalamus, amygdala, and posterior cingulate gyrus, involving ascending projections to brainstem nerve nuclei such as the NTS as central targets (Owens et al., 2024). Additionally, preclinical studies suggest that taVNS may regulate cortical excitability by modulating GABAergic inhibitory circuits and may alleviate neuroinflammatory damage by activating the cholinergic anti-inflammatory pathway (Gerges et al., 2025).

Accumulating evidence suggests preliminary safety and possible efficacy of taVNS in patients with DoC. A single session of taVNS can induce alterations in heart rate variability features (VLF/LF/SampEn) in DoC patients, which may help objective stratification and short-term outcome prediction (Li et al., 2025). Early studies using EEG have suggested that taVNS may enhance brain connectivity in MCS patients (Yifei et al., 2022). Zhou et al. reported in a randomized double-blind trial that 4 weeks of taVNS improved the level of consciousness in MCS patients but showed no significant effect in VS/UWS patients (Zhou et al., 2023). Gao et al. reported that long-term taVNS was associated with enhanced frontoparietal functional connectivity and increased CRS-R scores in UWS patients as monitored by fNIRS (Gao F. et al., 2025). Osińska et al. conducted a six-month longitudinal observation of a long-term UWS patient undergoing daily taVNS and found that the CRS-R score improved from 4–6 to 12–13 (reaching MCS/MCS+), accompanied by the emergence of *α* peaks in resting EEG, decreased HR, and increased HRV-HF. Follow-up indicated that the improvements were partially reversible, but such findings require further validation in RCTs (Osińska et al., 2022). RCTs investigating the long-term effects of taVNS and its combined application with tDCS have also been initiated (Vitello et al., 2023; Zhuang et al., 2023).

Existing clinical studies and systematic reviews indicate that taVNS exhibits overall good safety, with no significant difference in the incidence of adverse events compared with sham stimulation. Common adverse effects include ear tingling/discomfort, mild headache, or skin irritation, with no reports of serious adverse events causally related to taVNS. Therefore, taVNS demonstrates favorable tolerability and feasibility across various diseases and populations. However, standardized stimulation parameters across studies are lacking. The stimulation sites are mostly located at the cymba conchae or tragus regions, and studies in DoC populations typically use frequencies of 20–25 Hz, pulse widths of 100–500 μs, and intensities of 0.1–8 mA, with substantial heterogeneity. In DoC patients, individual calibration based on “perception/pain threshold” is often infeasible; thus, a fixed intensity is typically applied, with NCS-R monitoring used to assess tolerance. Overall, the “default frequency” in DoC research tends to be 20–25 Hz, with pulse width of 200–300 μs, and intensity set within a safe and tolerable range—maximally perceptible/activating yet avoiding pain or cardiovascular reactions (Zhuang et al., 2023; Zou et al., 2024). Peripheral nerve stimulation, including median nerve stimulation (MNS) and transcutaneous auricular vagus nerve stimulation (taVNS), offers new avenues for intervention in disorders of consciousness. Table 4 summarizes key information from relevant studies regarding stimulation targets, parameter settings, intervention duration, and clinical and neurophysiological outcomes.

**Table 4:** Summary of MNS and taVNS studies in disorders of consciousness.

Study/Author	Sample	Stimulation site	Parameters	Duration	Main outcomes
Wu et al. (2023)	Acute coma (TBI, 7–14 days post)	Right median nerve	RMNS, 8 h/day	2 weeks	Significantly higher consciousness recovery rate at 6 months; faster improvement in GCS, FOUR, CRS-R, GOSE
Yang et al. (2024)	Planned systematic review RCTs)	Median nerve	Various MNS protocols	Various	Protocol for systematic review and meta-analysis (not yet completed)
Xiong et al. (2023)	pDoC patients	Right wrist + left DLPFC	Combined MNS + rTMS	Multiple sessions	Combined stimulation enhanced CRS-R, GCS, SEP N20 amplitude/latency vs. single modality
Liu et al. (2022)	Post-traumatic DoC (120 patients)	Right median nerve	MNS + HBOT (simultaneous)	Multiple sessions	Synergistic effects; in-chamber protocol showed superior efficacy for GCS, EEG, evoked potentials
Zhou et al. (2023)	MCS and UWS	Auricular branch of vagus nerve	taVNS, 20–25 Hz	4 weeks	Improved consciousness in MCS; no significant effect in UWS
Gao et al. (2025)	UWS patients	Cymba conchae	Long-term taVNS	Extended duration	Enhanced frontoparietal functional connectivity (fNIRS); increased CRS-R
Osińska et al. (2022)	Long-term UWS patient	Auricular vagus nerve	Daily taVNS	6 months	CRS-R improved from 4–6 to 12–13 (MCS/MCS+); a peaks emerged in resting EEG; decreased HR, increased HRV-HF
Wang et al. (2022)	1856 patients (23 studies)	Median nerve	Various MNS protocols	Various	Improved GCS, EEG grading, cerebral blood flow; dose–response relationship
Cooper et al. (1999)	30 coma patients (TBI)	Right median nerve	MNS	2 weeks	Improved GCS; shortened hospital stay
Peri et al. (2001)	Coma patients	Median nerve	MNS	3 months follow-up	Pilot study showing potential benefits
Jia et al. (2022)	5 TBI coma patients	Right median nerve	RMNS	2 weeks	CSF miRNA changes related to neuroprotection
Sun et al. (2024)	TBI animal model	Median nerve	MNS, 20 Hz	Multiple sessions	Reduced TACR1, inhibited NF-κB and CCL7 in microglia
Yu et al. (2017)	Single VS patient	Auricular vagus nerve	taVNS, 25 Hz	Several weeks	First case report; CRS-R improved; fMRI monitored
Noé et al. (2020)	DoC cohort	Auricular vagus nerve	taVNS, 25 Hz	Multiple sessions	Feasibility and safety confirmed; MCS responded better
Yifei et al. (2022)	DoC patients	Auricular vagus nerve	taVNS, 20–25 Hz	Multiple sessions	Improved brain connectivity; δ↓ β↑ in MCS
Yu et al. (2021)	DoC patients	Auricular vagus nerve	taVNS, 25 Hz	Multiple sessions	Cerebral hemodynamic changes; auditory function predicted response

MNS, median nerve stimulation; RMNS, right median nerve stimulation; taVNS, transcutaneous auricular vagus nerve stimulation; TBI, traumatic brain injury; DoC, disorders of consciousness; pDoC, prolonged disorders of consciousness; MCS, minimally conscious state; UWS, unresponsive wakefulness syndrome; CRS-R, Coma Recovery Scale-Revised; GCS, Glasgow Coma Scale; FOUR, Full Outline of UnResponsiveness; GOSE, Glasgow Outcome Scale-Extended; RCT, randomized controlled trial; RR, risk ratio; SEP, somatosensory evoked potential; HBOT, hyperbaric oxygen therapy; fNIRS, functional near-infrared spectroscopy; HRV-HF, heart rate variability high frequency.

### 1.7 Transcranial focused ultrasound (tFUS)

tFUS is an emerging non-invasive neuro-regulation technology that utilizes low-intensity ultrasound waves (typically ranging from 0.25 to 0.5 megahertz) to transmit through the skull to the brain tissue, enabling precise regulation of specific brain regions (Ho et al., 2025). The interaction between ultrasound waves and neural tissues involves multiple physical mechanisms. Among them, the mechanical effect is the main mechanism, where the acoustic radiation force causes micro-mechanical deformation of the nerve cell membrane, leading to changes in membrane capacitance and conformational changes in voltage-gated ion channels (Shi Y. et al., 2025). The thermal effect is relatively minor under low-intensity parameters but must be considered in terms of safety (Antoniou et al., 2024). Additionally, the cavitation effect can cause the microbubbles within the cell membrane lipid bilayer to vibrate, potentially affecting membrane properties and cell signal transduction (Javid et al., 2023; Yang et al., 2025). The core advantage of tFUS lies in its unique spatial focusing ability. Through single-element transducers or multi-element phased array technology, ultrasound waves can form a highly precise focused area within the skull with a diameter of only a few millimeters, enabling non-invasive positioning of deep brain structures (Davidson et al., 2025). This feature renders tFUS particularly valuable in the treatment of DoC—unlike TMS and tDCS, which are limited to superficial cortical stimulation, tFUS can directly modulate subcortical nuclei such as the thalamus, which are essential for consciousness, without requiring invasive surgery as in Deep Brain Stimulation (DBS) (Yang et al., 2025). Based on energy intensity, transcranial ultrasound can be classified into two main types. Low-Intensity Focused Ultrasound (LIFU/LITUS) is used for reversible neuromodulation and is the primary modality employed in current DoC research, while High-Intensity Focused Ultrasound (HIFU) achieves tissue ablation through thermal effects but, due to its irreversibility, is unsuitable for neuromodulation in DoC (Darrow, 2019). Clinical translation of LIFU in DoC began with the first human case report by Monti et al. The researchers applied tFUS stimulation (frequency 650 kHz, pulse repetition frequency 100 Hz, spatial-peak pulse-average intensity 720 mW/cm^2^) to the thalamus of a 25-year-old male patient with acute DoC. The patient’s CRS-R score improved from 14 to 15 before treatment to 13–17 after stimulation; within 3 days of tFUS, he regained full language comprehension and reliable communication ability, and by day 5, he attempted walking (Monti et al., 2016). Cain and colleagues subsequently conducted a series of systematic studies. Among 11 acute DoC patients, a single tFUS session significantly increased CRS-R scores, with four patients showing diagnostic category improvement (Cain et al., 2022). In another study involving 10 patients with chronic DoC, two treatments administered at intervals of 1 week resulted in improvement in the condition of 4 patients, and the overall CRS-R score showed a significant linear upward trend (Cain et al., 2021). Regarding safety, in four clinical studies involving a total of 24 DoC patients, no serious adverse events were reported. Minor adverse reactions observed in other neurological diseases, such as headache, neck pain, scalp tingling, and drowsiness, usually resolved spontaneously within 24 h.

The molecular mechanisms involved in the regulation of neural activity by tFUS span multiple levels. At the membrane level, preclinical studies indicate that the mechanical stress caused by ultrasound can activate mechanosensitive ion channels. Sorum et al. demonstrated that ultrasound can directly activate mechanosensitive TRAAK K^+^ channels through the lipid membrane in experimental models (Sorum et al., 2021). Yoo et al. found in preclinical experiments that tFUS can activate mechanosensitive calcium channels, induce calcium accumulation in cortical neurons, and amplify the effect through ion channels to enhance neuronal excitability (Yoo et al., 2022). At the synaptic and network level, preclinical studies further suggest that tFUS can regulate neurotransmitter release and neural oscillations. For example, experimental work has shown that tFUS enhances dopamine release in the striatum, indicating its potential to regulate arousal-related neurotransmitter systems (Olaitan et al., 2025). By contrast, in human studies, Legon et al. reported that thalamic tFUS significantly modulated thalamo-cortical functional connectivity as measured by EEG and functional magnetic resonance imaging (fMRI), a finding particularly relevant to DoC, in which disruption of thalamo-cortical connectivity represents a core pathological feature (Legon et al., 2018).

### 1.8 Acupuncture therapy

As a core treatment modality of traditional Chinese medicine (TCM) (Ma Q. et al., 2025), acupuncture is widely applied in China as an adjunctive therapy for DoC. Its theoretical foundation lies in stimulating specific acupoints to regulate the neuro-endocrine-immune network, modulate the function of the neurovascular unit (Shi A. et al., 2025), enhance cerebral blood perfusion in ischemic regions, and improve the function of damaged neurons (Chang et al., 2018). A systematic review conducted by Huang et al. included 17 randomized controlled trials (RCTs), involving a total of 1,208 stroke patients with cerebral infarction, and provided aggregated evidence suggesting possible benefit of acupuncture therapy. The meta-analysis indicated that the improvement in GCS scores in the acupuncture group was significantly greater than that in the non-acupuncture group (mean difference = 1.45, 95% confidence interval 0.94 to 1.97, *p* < 0.0001). Group analysis showed that the combination of acupuncture and drug treatment was significantly superior to drug treatment alone (mean difference = 1.81, 95% confidence interval 1.24 to 2.39, *p* < 0.0001); likewise, the combination of acupuncture and rehabilitation intervention measures (including music therapy and repetitive transcranial magnetic stimulation) was more effective than simple rehabilitation treatment alone (mean difference = 2.48, 95% confidence interval 1.42 to 3.53, *p* < 0.0001). Notably, no significant difference was observed between acupuncture alone and either pharmacotherapy or rehabilitation therapy alone, suggesting that acupuncture may exert its optimal effects through synergistic interactions with other treatments (Huang et al., 2022). Regarding arousal-promoting effects, four studies reported that the resuscitation rate was significantly higher in the acupuncture plus drug group than in the drug-only group (RR = 1.68, 95% CI 1.30–2.18, *p* < 0.001). Other clinical studies have also suggested that acupuncture may improve behavioral manifestations in patients with DoC (Wu et al., 2024).

The selection of acupoints in acupuncture therapy for DoC follows certain regularities. The most frequently used acupoints in the included studies were Shuigou (GV26), Neiguan (PC6), Baihui (GV20), and Sanyinjiao (SP6) (Guo et al., 2022). The selection of these acupoints is closely related to the TCM theory of “restoring consciousness by opening the orifices.” Specifically, Shuigou and Baihui are located on the Governor Vessel and are believed to have effects of awakening the mind and opening the orifices; Neiguan, belonging to the Pericardium meridian, regulates the mind; and Sanyinjiao, the intersection point of the Liver, Spleen, and Kidney meridians, nourishes yin, enriches blood, and calms the spirit (Xun et al., 2025). Modern mechanistic studies on acupuncture-induced arousal have revealed the involvement of multiple pathways. In animal experiments, Tang et al. (2025) found that acupuncture may activate dopaminergic neurons and P2RX7 receptors in the ventral periaqueductal gray matter, and modulate the expression of neural projection-related genes (P2rx7, P2rx3, Trpv1, etc.) and dopamine D3 receptors. In addition, animal studies and limited clinical observations suggest that acupuncture or electroacupuncture during the post-ischemic hypoxic phase may attenuate inflammation, reduce oxidative stress, and stabilize intracellular calcium homeostasis, thereby potentially reducing neuronal apoptosis; it may also promote the release of neurotrophic factors to facilitate functional recovery (Chen and Hsieh, 2020). In terms of safety, only two studies reported adverse events, both of which involved mild subcutaneous hematoma that resolved with simple management; no severe adverse reactions were observed. However, the current evidence has notable limitations. Many included studies carry a high risk of bias, mainly due to inadequate descriptions of randomization methods, lack of allocation concealment, and absence of blinding of outcome assessors. Furthermore, most studies were published in China, raising concerns of regional and publication bias. Therefore, although acupuncture is widely used as an adjunctive therapy for DoC in Chinese clinical practice, its efficacy requires further validation through rigorously designed, high-quality, multicenter RCTs.

### 1.9 Phototherapy and circadian rhythm regulation

#### 1.9.1 Transcranial photobiomodulation (tPBM)

tPBM is a noninvasive neuromodulation technique that utilizes low-intensity red to near-infrared light, with wavelengths ranging from 600 to 1,300 nm, most commonly 810 nm, to penetrate the skull and act directly on brain tissue (Motsenyat et al., 2025). Its core mechanism involves cytochrome c oxidase (CCO), a component of mitochondrial respiratory chain complex IV, which absorbs photons leading to the dissociation of inhibitory nitric oxide, thereby enhancing mitochondrial activity and promoting ATP biosynthesis (Hamblin, 2016; Liu et al., 2026). Given the high mitochondrial density of brain tissue, tPBM may exert neuroprotective effects by modulating cerebral metabolism and blood flow. Both preclinical and clinical studies have confirmed the neuroprotective effects of tPBM in traumatic brain injury (TBI). In a mouse model of moderate-to-severe TBI, the Hamblin group demonstrated that a single transcranial irradiation with red light (660 nm) or near-infrared light (810 nm) administered 4 h post-injury significantly improved neurological severity scores and reduced lesion volume; moreover, 3 consecutive days of tPBM treatment were more effective than a single exposure in improving motor and memory function, reducing neuronal degeneration and apoptosis in the lesion area, and promoting neurogenesis, synaptogenesis, and BDNF expression in the dentate gyrus and subventricular zone (Montazeri et al., 2021). In a small clinical study with chronic severe traumatic brain injury, tPBM was associated with improved cerebral blood flow velocity and neuropsychological function (Naeser et al., 2011). However, direct clinical evidence of tPBM in DoC remains extremely limited. Light undergoes exponential attenuation as it passes through the skull and brain tissue, so the number of photons reaching the deep structures of the brain is very limited, which poses a challenge in regulating the thalamic nuclei (a key area closely related to consciousness) (Tedford et al., 2015; Tittelmeier et al., 2025). The parameters in dementia research vary greatly (wavelength 630–1,100 nanometers, power density 0.01–50 milliwatts per square centimeter, treatment time ranging from a few minutes to several tens of minutes). Currently, the optimal treatment plan has not been established. However, due to its non-invasive, safe and easy-to-use characteristics, tPBM is still regarded as a promising adjunctive treatment method and is worthy of further exploration in the field of consciousness disorders (Zhang X. et al., 2025).

#### 1.9.2 Circadian rhythm and light therapy

In patients with delayed encephalopathy following severe brain injury, disruption of the circadian rhythm is a common problem, which manifests as abnormal body temperature rhythms, abnormal heart rate variability, and abnormal sleep–wake cycles (Wislowska et al., 2017). Studies have shown that patients with DoC who have more accurate clinical diagnoses typically exhibit more complete circadian rhythms of melatonin and body temperature, more obvious circadian components in wrist activity records, and more regular sleep–wake cycles (Cruse et al., 2013). These findings suggest a potential association between circadian rhythm stability and consciousness recovery.

Angerer et al. conducted the first systematic assessment of the impact of simulated natural daylight on the circadian rhythms of DoC patients. A total of 17 patients were exposed to either standard ward lighting or simulated dynamic daylight conditions for 7 to 8 days. The results showed that under dynamic daylight conditions, the body temperature cycles of 11 patients were close to 24 h, with increased rhythm strength and improved daytime stability (Angerer et al., 2022). Additional exploratory observations have also been reported. Yelden et al. applied blue-light-enriched glasses delivering short-wavelength light to 8 DoC patients three times daily for 1 h over 1 week, with two patients changing diagnostically from VS/UWS to MCS. Another interventional study involving 17 DoC patients implemented combined melatonin and blue-light therapy for 5 weeks and reported improvements in sleep–wake patterns (Yelden et al., 2022). A plausible interpretation is that light acts as a major timing cue for circadian regulation. Through specialized retinal photoreceptors and the suprachiasmatic nucleus (SCN), light can modulate melatonin secretion and core body temperature rhythm. Appropriate light exposure may help entrain the biological clock and support sleep–wake cycle organization, which could in turn be relevant to maintaining arousal and consciousness (Gooley et al., 2011; Lucas et al., 2014).

### 1.10 Hyperbaric oxygen therapy (HBOT)

HBOT is a treatment method in which patients inhale pure oxygen in an environment with pressure above normal atmospheric pressure (usually 0.2 megapascals, equivalent to 2 absolute atmospheres). It is widely used in China as an adjunctive treatment for DoC (Bin-Alamer et al., 2024). The theoretical basis of this approach lies in increasing the oxygen partial pressure in the blood and tissues, in order to enhance the oxygen supply to the hypoxic brain regions, thereby promoting neural plasticity and functional recovery. The neuro-regulatory mechanism of hyperbaric oxygen therapy involves multiple aspects. Firstly, hyperbaric oxygen enhances mitochondrial generation and function by upregulating Bcl-2, downregulating Bax, and promoting ATP production. Additionally, it activates TRAF3, Wnt-3, and VEGF/ERK signaling pathways to promote neurogenesis, while reducing inflammatory cytokines such as TNF-*α* and IL-6 (Huang et al., 2025; Yang et al., 2025). A retrospective cohort study conducted by Li et al. involved 484 patients with DoC. The results showed that hyperbaric oxygen therapy was associated with improved GCS scores and Nanjing Persistent Vegetative State Scale scores, with a reported total effective rate of 67.1%. Multivariate analysis identified several factors associated with better prognosis, including intervention time ≤ 7 days, more treatment sessions, higher pretreatment GCS score, and traumatic etiology (Li S. et al., 2024). Another study involving 255 pediatric DoC patients similarly suggested that early HBOT intervention and baseline neurological status may be associated with recovery (Zhao et al., 2025). Combined therapeutic strategies may also be relevant. Liu et al. randomized 120 patients with post-traumatic DoC into three groups and found that performing MNS simultaneously during HBOT was associated with better outcomes than MNS applied after HBOT, with gradient improvements observed in GCS scores, EEG findings, and evoked potentials (Liu et al., 2022).

### 1.11 Spinal cord stimulation (SCS)

SCS is a neuromodulation technique in which electrodes are implanted in the cervical epidural space to deliver electrical pulse stimulation to the spinal cord nerves (Rubin, 2025). The arousal-promoting mechanism of SCS is generally thought to involve activation of the reticulo-thalamo-cortical pathway and a related increase in cerebral blood flow (Piedade et al., 2023). Early studies by Yamamoto et al. found that SCS increased whole-brain blood flow in MCS patients by more than 22% during stimulation compared with the pre-stimulation period. In recent years, short-term spinal cord stimulation (st-SCS) has emerged as a minimally invasive alternative and has shown preliminary progress in the field of DoC. In a pilot study by Zhuang et al., 31 patients with chronic pDoC underwent 2 weeks of st-SCS treatment, resulting in a significant increase in CRS-R scores. Group analysis suggested that patients with MCS − may show greater improvement than those with VS/UWS or MCS+. Regarding stimulation frequency, both 5 Hz and 70 Hz were associated with recovery-related improvement, although 70 Hz showed a larger increase in CRS-R score 1 week after treatment (Zhuang et al., 2022; Zhuang et al., 2024). Studies on spinal cord stimulation therapy (st-SCS) for DoC caused by primary brainstem hemorrhage have also shown similar favorable results. Among 14 patients, more than 70% of them had a CRS-R score increase of at least 2 points after 2 weeks of treatment, and the rate of clinical diagnosis improvement was 50% (Huang et al., 2023b). Sun et al. evaluated 27 post-SCS DoC patients using EEG power spectrum and event-related potentials and found that those who improved in consciousness exhibited shortened P300 latency and increased amplitude, suggesting that SCS modulates cortical electrical activity associated with consciousness. The “ABCD” EEG model proposed by the researchers can be used for preoperative evaluation, with type B or C EEG patterns predicting better SCS efficacy, thereby improving surgical success rates (Sun et al., 2024). TMS-EEG studies have further revealed the cortical modulation effects of SCS. After a single session of SCS, changes were observed in global field potentials and the perturbational complexity index in DoC patients, with more pronounced cortical activity changes in MCS patients. Follow-up results indicated that 50% of MCS patients and 20% of UWS patients exhibited more conscious behaviors 6 months after SCS (Wang et al., 2023). Long-term outcome analysis by Yang et al. demonstrated that SCS is a feasible therapeutic approach to promote arousal in DoC patients, particularly in those with MCS and traumatic etiologies. The future direction for enhancing neuromodulation efficacy lies in developing individualized stimulation parameters and closed-loop stimulation strategies based on patient-specific characteristics (Yang et al., 2022). The summary is shown in Table 5.

**Table 5:** Summary of other neuromodulation techniques in disorders of consciousness.

Study/Author	Technique	Sample	Parameters	Duration	Main outcomes
Monti et al. (2016)	tFUS	Single acute DoC patient (25 y/o male)	650 kHz, PRF 100 Hz; thalamus	Single session	CRS-R improved 14–15 to 13–17; regained language comprehension within 3 days; attempted walking by day 5
Cain et al. (2022)	tFUS	11 acute DoC patients	Thalamic stimulation	Single session	Significant CRS-R increase; 4 patients showed diagnostic category improvement
Huang et al. (2022)	Acupuncture	1,208 post-stroke DoC (17 RCTs)	Various acupoints (GV26, PC6, GV20, SP6)	Various	GCS improvement MD = 1.45 (P < 0.0001); acupuncture+drugs RR = 1.68 for resuscitation rate
Naeser et al. (2011)	tPBM	2 chronic severe TBI patients(case reports)	810 nm NIR light	Multiple sessions	Improved cerebral blood flow velocity and neuropsychological function
Angerer et al. (2022)	Light therapy	17 DoC patients	Simulated dynamic daylight	7–8 days	11 patients showed ~24-h body temperature cycles; increased rhythm strength
Yelden et al. (2022)	Blue-light therapy	8 DoC patients	Blue-light-enriched glasses, 3x1h/day	1 week	2 patients improved from UWS to MCS
Li et al. (2024)	HBOT	484 DoC patients (retrospective)	0.2 MPa (2 ATA)	Multiple sessions	Total effective rate 67.1%; improved GCS and Nanjing PVS Scale; early intervention better outcomes
Zhao et al. (2025)	HBOT	255 pediatric DoC patients	Standard HBOT protocol	Multiple sessions	Early intervention and baseline neurological status predicted recovery
Zhuang et al. (2022) and Zhuang et al. (2024)	st-SCS	31 chronic pDoC patients	Cervical epidural, 5 Hz and 70 Hz	2 weeks	Significant CRS-R increase; showed greater improvement; 70 Hz effects persisted 1 week post-treatment
Huang et al. (2023b)	st-SCS	14 primary brainstem hemorrhage DoC	Cervical SCS	2 weeks	>70% showed CRS-R increase; ≥2 points; 50% diagnostic improvement rate
Sun et al. (2024)	SCS	27 post-SCS DoC patients	Cervical SCS	Variable	Improved patients showed shortened P300 latency, increased amplitude; ABCD EEG model for prediction
Wang et al. (2023)	SCS + TMS-EEG	DoC patients	Cervical SCS	Single session	50% MCS and 20% UWS showed more conscious behaviors at 6-month follow-up
Yang et al. (2022)	SCS (long-term)	DoC patients	Cervical SCS	6 months follow-up	SCS is feasible for promoting arousal in DoC; MCS and traumatic etiology associated with better outcomes; identified related prognostic factors

tFUS, transcranial focused ultrasound; tPBM, transcranial photobiomodulation; HBOT, hyperbaric oxygen therapy; SCS, spinal cord stimulation; st-SCS, short-term spinal cord stimulation; DoC, disorders of consciousness; pDoC, prolonged disorders of consciousness; MCS, minimally conscious state; UWS, unresponsive wakefulness syndrome; TBI, traumatic brain injury; CRS-R, Coma Recovery Scale-Revised; GCS, Glasgow Coma Scale; PRF, pulse repetition frequency; NIR, near-infrared; ATA, absolute atmospheres; PVS, persistent vegetative state; MD, mean difference; RR, risk ratio; P300, event-related potential P300 component; EEG, electroencephalography; TMS-EEG, transcranial magnetic stimulation combined with electroencephalography; MPa, megapascals.

### 1.12 The mesocircuit hypothesis as an integrative model for neuromodulation in DoC

While the preceding sections have summarized the evidence for individual neuromodulation modalities, a critical synthesis is needed to understand why these diverse techniques produce heterogeneous and often conflicting outcomes, and how their mechanisms can be unified within an established theoretical framework. The mesocircuit hypothesis, originally proposed by Schiff (2010), provides such a unifying framework. This model posits that severe brain injuries—regardless of etiology—produce widespread deafferentation and neuronal loss, leading to reduced excitatory drive from the central thalamus to the frontal cortex and striatum. The consequent loss of medium spiny neuron (MSN) output releases tonic inhibition from the globus pallidus interna (GPi) onto the central thalamus, creating a self-reinforcing cycle of anterior forebrain downregulation (Schiff, 2010; Schiff, 2023). This circuit-level dysfunction is not limited to a single anatomical pathway but rather affects the entire cortico-striatopallidal-thalamocortical loop, which constitutes the core substrate of forebrain arousal and executive function (Dutta et al., 2025). The anterior forebrain mesocircuit is also intimately connected with the frontoparietal network (FPN), which has been independently identified as a key neural correlate of consciousness through functional neuroimaging studies (Demertzi et al., 2015; Monti et al., 2015). Disrupted FPN connectivity is consistently observed in UWS patients, while partial preservation of this network characterizes MCS (Bai et al., 2024; Rosanova et al., 2018). Thus, the mesocircuit and FPN frameworks converge to identify a shared anatomical substrate—the thalamocortical-frontoparietal axis—whose functional integrity determines the level of consciousness and, critically, the capacity to respond to neuromodulatory interventions.

Crucially, the mesocircuit model suggests, at a theoretical level, that interventions capable of augmenting excitatory neurotransmission at any node within this loop may contribute to restoring the functional balance of the circuit, although direct causal evidence from human studies remains limited (Schiff, 2023; Edlow et al., 2021). This prediction aligns with the observed convergence of therapeutic effects across modalities that ostensibly operate through distinct biophysical mechanisms. Direct cortical stimulation techniques (rTMS, tDCS) enhance excitability of the frontal cortex—a key cortical node of the mesocircuit—and can propagate effects to connected subcortical structures via corticostriatal and corticothalamic projections (Avalos-Alais et al., 2026). tFUS, uniquely among non-invasive modalities, can directly target the central thalamus—the bottleneck of the mesocircuit—and has been reported in small studies to modulate thalamo-cortical functional connectivity and enhance striatal dopamine release; these findings are consistent with the hypothesis that such changes may relieve GPi-mediated thalamic inhibition, though this mechanism requires confirmation in larger human studies (Olaitan et al., 2025; Legon et al., 2018). Bottom-up peripheral stimulation techniques activate the mesocircuit through ascending pathways: MNS engages the ascending reticular activating system (ARAS) via somatosensory afferents projecting through the brainstem reticular formation to the central thalamus (Feng and Du, 2016); taVNS activates the locus coeruleus-norepinephrine system, which provides diffuse excitatory neuromodulation to cortical and thalamic targets (Tong et al., 2025; Chen et al., 2023); and SCS activates the reticulo-thalamo-cortical pathway, directly increasing whole-brain cerebral blood flow (Piedade et al., 2023). Thus, each modality can be understood as targeting a specific entry point into a shared neural circuit. The therapeutic outcome may be substantially influenced by the degree of residual circuit integrity, rather than by the modality itself—a hypothesis consistent with current data but not yet definitively established.

**Figure 1:**
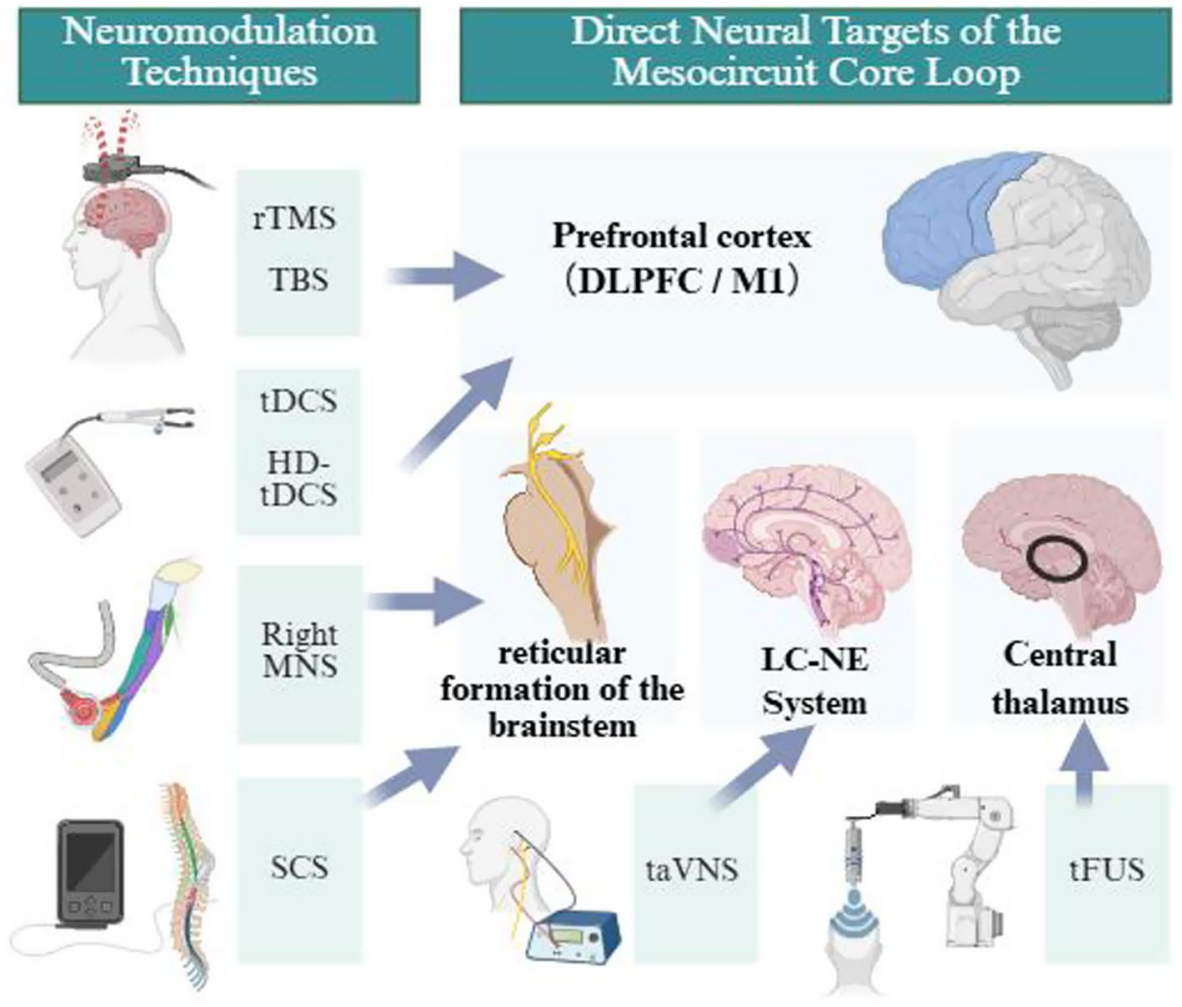
Schematic overview of noninvasive and minimally invasive neuromodulation techniques and their direct neural targets within the mesocircuit core loop. The left panel illustrates several neuromodulation approaches, including rTMS (repetitive transcranial magnetic stimulation), TBS (theta burst stimulation), tDCS (transcranial direct current stimulation), HD-tDCS (high-definition transcranial direct current stimulation), right MNS (right median nerve stimulation), and SCS (spinal cord stimulation). The right panel depicts the corresponding direct neural targets of the mesocircuit core loop, comprising the prefrontal cortex (DLPFC, dorsolateral prefrontal cortex / M1, primary motor cortex), the reticular formation of the brainstem, the LC-NE system (locus coeruleus–norepinephrine system), and the central thalamus. Additionally, taVNS (transcutaneous auricular vagus nerve stimulation) and tFUS (transcranial focused ultrasound stimulation) are shown to engage these targets. Arrows indicate the putative pathways linking each neuromodulation technique to its respective neural target.

This integrative perspective provides a mechanistic explanation for one of the most consistent findings across the neuromodulation literature: the differential treatment responsiveness of MCS versus UWS patients. The mesocircuit model hypothesizes that clinical response may depend on a minimum threshold of residual circuit integrity upon which neuromodulatory inputs can act (Schiff, 2010). In MCS patients, the mesocircuit is partially preserved—MSN populations retain some excitatory drive, thalamocortical projections remain partially functional, and frontoparietal connectivity is diminished but not abolished (Ma et al., 2024; Bai et al., 2024). This residual infrastructure provides a substrate for neuromodulation to amplify subthreshold activity back above the critical level necessary for conscious behavior. In contrast, UWS patients typically exhibit more severe disruption—extensive neuronal loss, marked GPi-mediated thalamic suppression, and profoundly reduced frontoparietal connectivity (Comanducci et al., 2020; Rosanova et al., 2018)—leaving insufficient residual circuitry for neuromodulatory inputs to engage. This prediction is supported by the consistent observation across rTMS (Liu et al., 2025; Legostaeva et al., 2019), tDCS (Thibaut et al., 2023; Hermann et al., 2020), taVNS (Zhou et al., 2023), and SCS (Yang et al., 2022) studies that MCS patients show significantly greater behavioral improvements than UWS patients. Furthermore, within the UWS category, emerging evidence suggests that patients with electroencephalographic signatures of partially preserved mesocircuit function (e.g., higher baseline alpha power, detectable P300 responses, or preserved PCI values) may represent a subgroup with latent recovery potential that could be unmasked by neuromodulation (He et al., 2020; Thibaut et al., 2018). This observation underscores the necessity of moving beyond categorical diagnosis toward biomarker-guided stratification to identify the true target population for each intervention. Figure 1 provides a schematic synthesis of the convergent circuit-level mechanisms by which diverse neuromodulation modalities promote consciousness recovery within the anterior forebrain mesocircuit framework.

### 1.13 Stimulation parameter heterogeneity, etiology, and chronicity as determinants of conflicting outcomes

A major source of conflicting results across the neuromodulation literature lies in the marked heterogeneity of stimulation parameters, patient chronicity, and etiological composition of study samples—factors that interact in complex ways to determine treatment outcomes but have not been sufficiently analyzed in an integrated manner. Within the tES domain, studies vary substantially in current intensity (1–4 mA), electrode montage (conventional bifocal pad electrodes vs. high-definition 4 × 1 ring arrays), stimulation duration (single 20-min session vs. multiple weeks of daily sessions), polarity configuration (unilateral anodal vs. bilateral sequential), and target site (left DLPFC, bilateral DLPFC, M1, precuneus). The multicenter RCT by Thibaut et al. (2023) demonstrated no short-term superiority of anodal tDCS over sham stimulation using a standard left DLPFC protocol with conventional electrodes (2 mA, 20 min, 5 sessions), yet subgroup analyses at three-month follow-up revealed significant CRS-R improvements specifically in MCS and traumatic-etiology patients. This temporally delayed, subgroup-specific effect raises critical questions about whether the initial negative finding reflects true treatment inefficacy or, alternatively, the masking of genuine effects by the inclusion of non-responsive patient subpopulations (UWS patients, non-traumatic etiologies) in an underpowered overall analysis. Furthermore, the use of conventional tDCS with large sponge electrodes (typically 5 × 7 cm) results in highly diffuse current distribution, with finite element modeling studies showing that less than 50% of the injected current may reach the intended cortical target (Aberra et al., 2023)—a problem substantially compounded in DoC patients who frequently present with skull defects from decompressive craniectomy, cerebral edema, enlarged ventricles, and cortical atrophy, all of which dramatically alter current pathways (Ma et al., 2024). HD-tDCS, which uses a 4 × 1 ring montage with smaller electrodes (<12 mm diameter), achieves markedly improved spatial focality and may overcome these limitations. Preliminary studies applying HD-tDCS to the precuneus in chronic DoC patients have reported significant CRS-R improvements and enhanced mismatch negativity responses (Wang et al., 2020), suggesting that improved target engagement through focal stimulation may substantially enhance therapeutic efficacy, though these findings require replication in larger, sham-controlled trials.

Similar parameter heterogeneity pervades the rTMS literature, where the divergence between studies reporting significant CRS-R improvements with 10–20 Hz DLPFC stimulation over 4 weeks (Liu et al., 2025; Fan et al., 2022) versus null behavioral findings with only 5 sessions targeting M1 (Cincotta et al., 2015; He et al., 2018) underscores that cumulative dose (total number of pulses and sessions), stimulation frequency, and target selection relative to individual lesion topography are likely critical determinants of efficacy. A meta-analysis of sham-controlled RCTs confirmed the superiority of DLPFC-targeted rTMS for DoC (Gong et al., 2025), but the included studies employed stimulation durations ranging from 5 to 40 sessions, frequencies from 5 to 20 Hz, and intensities from 80 to 120% of resting motor threshold—a range of parameters that may span the boundary between subtherapeutic and effective doses. The observation that 5 sessions of 20 Hz rTMS targeting M1 produced detectable neuroimaging changes (enhanced functional connectivity) without corresponding behavioral improvement (Cincotta et al., 2015; He et al., 2018) is particularly instructive: it suggests that short-duration protocols may induce transient neuroplastic changes that are insufficient in magnitude or duration to cross the clinical threshold for behavioral change, but that could potentially become clinically meaningful with extended treatment. This dose-dependent hypothesis is supported by the positive results observed with longer protocols (10–40 sessions) (Liu et al., 2025; Wan et al., 2024a) and by the dose–response relationship confirmed in MNS meta-analyses, where longer treatment durations were associated with greater GCS improvements (Wang et al., 2022). Future rTMS studies should systematically investigate dose–response relationships using adaptive trial designs, and employ computational electric field modeling or neuronavigation-guided targeting to optimize coil placement for individual patients, accounting for the substantial inter-individual variability in cortical anatomy and lesion patterns that is characteristic of the DoC population (Ma et al., 2024).

Beyond parameter selection, two patient-level variables—etiology and chronicity—emerge as critical moderators of treatment response that have been insufficiently stratified in most published studies. Regarding etiology, TBI patients consistently demonstrate better neuromodulation responses compared to those with anoxic or vascular etiologies across multiple modalities: the Thibaut et al. tDCS trial found significant effects only in the traumatic subgroup (Thibaut et al., 2023); MNS meta-analyses show higher consciousness recovery rates in TBI (Wu et al., 2023; Yang et al., 2025); SCS long-term outcome analyses identify traumatic etiology as an independent predictor of favorable response (Yang et al., 2022); and HBOT retrospective studies confirm that traumatic etiology is associated with better prognosis (Li S. et al., 2024). This etiology-dependent response pattern likely reflects fundamental differences in the neuropathology of injury: TBI predominantly causes diffuse axonal injury and white matter disconnection, which disrupts circuit function while potentially preserving neuronal cell bodies that remain viable targets for reactivation; in contrast, anoxic injury produces widespread cortical and subcortical gray matter necrosis, particularly affecting the metabolically vulnerable hippocampus, thalamus, and basal ganglia—core components of the mesocircuit—leaving fewer residual neurons to respond to neuromodulatory input (Edlow et al., 2021; Giacino et al., 2014). Vascular etiologies present intermediate patterns depending on lesion location and extent. Regarding chronicity, a clear temporal gradient of treatment responsiveness is suggested by the available evidence: early intervention (≤7 days post-injury) yields better HBOT outcomes than delayed treatment (Li S. et al., 2024); MNS applied 7–14 days post-TBI achieves significant 6-month recovery rates (Wu et al., 2023); while chronic DoC patients (>12 months) show substantially attenuated responses across modalities (Yang et al., 2022; Martens et al., 2018). This chronicity effect likely reflects the progressive consolidation of maladaptive circuit states—including Wallerian degeneration of disconnected axons, synaptic loss, and establishment of aberrant inhibitory networks—that progressively reduce the reserve of recruitable circuitry. However, the persistence of neuroplasticity even in chronic DoC is evidenced by individual responders to late-stage interventions (Gao F. et al., 2025; Osińska et al., 2022), emphasizing that chronicity should inform treatment expectations and protocol intensity rather than serve as an absolute exclusion criterion.

Table 6 synthesizes the interrelationships among stimulation parameters, patient-level moderators, and clinical outcomes across the major neuromodulation modalities reviewed in this manuscript, providing a structured framework for understanding how specific parameter variations contribute to the observed heterogeneity in results.

**Table 6:** Interrelationships among stimulation parameters, patient moderators, and clinical outcomes across neuromodulation modalities for DoC.

Modality	Key parameter variation	Parameter range across studies	Etiology effect	Chronicity effect	Impact on outcome and mechanistic interpretation	References
rTMS	Stimulation frequency	5–20 Hz; 80–120% RMT	TBI > anoxic; post-traumatic VS responded to M1 20 Hz	4 weeks > 5 sessions; subacute > chronic	10–20 Hz DLPFC over 4 weeks: significant CRS-R improvement and enhanced TMS-EEG connectivity. 5 sessions at M1: neuroimaging changes only, no behavioral effect. Suggests cumulative dose threshold for clinical translation.	Gong et al. (2025), Liu et al. (2025), Fan et al. (2022), Wan et al. (2024a), Cincotta et al. (2015) and He et al. (2018)
rTMS	Target site selection	DLPFC, M1, parietal cortex, angular gyrus, cerebellum	Optimal target may be individualized; frontal targets vulnerable in TBI	Multicenter personalized trial ongoing	DLPFC has strongest meta-analytic support. Parietal targets effective in some studies. Angular gyrus: MCS improved, UWS no change. Suggests that optimal target depends on individual lesion topography and residual network integrity.	Gong et al. (2025), Liu et al. (2025), Wan et al. (2024a), and Legostaeva et al. (2019)
tDCS	Electrode montage and focality (Martens et al., 2018; Zhang X. et al., 2021)	Conventional (5 × 7 cm pads) vs. HD-tDCS (4 × 1 ring, <12 mm); 1–4 mA	Traumatic subgroup: significant 3-month improvement; non-traumatic: NS	Chronic MCS: home-based 4-week protocol showed moderate benefit	Conventional tDCS: no overall short-term superiority vs. sham in multicenter RCT; subgroup effects in MCS and TBI at follow-up. HD-tDCS: improved spatial focality, preliminary positive results at precuneus. Diffuse current in conventional montage may explain null overall results.	Thibaut et al. (2023), Martens et al. (2018), Wang et al. (2020), Guo et al. (2019), Aberra et al. (2023), and Ma et al. (2024)
tDCS	Number of sessions and follow-up duration	Single session to 80 sessions; follow-up 0–24 weeks	TBI patients show delayed (3-month) response not visible short-term	Subacute to chronic; long-term protocols (80 sessions) effective for both MCS and UWS	Single-session studies detect EEG changes but not behavioral improvement. Multi-session protocols (10–80 sessions) show cumulative behavioral gains. Delayed effects at 3-month follow-up suggest neuroplastic consolidation requiring extended treatment.	Thibaut et al. (2023), Martens et al. (2018), Hermann et al. (2020), and Zhang et al. (2021)
MNS	Stimulation duration and laterality	Right-sided; 8 h/day × 2 weeks; 7–14 days post-injury	Evidence in acute TBI; limited data in non-traumatic DoC	Acute phase (7–14 days post-TBI): RR = 1.36; dose–response: longer duration → greater GCS gain	Right MNS activates contralateral somatosensory cortex and ARAS. Early acute intervention maximizes benefit. Dose–response relationship confirmed in meta-analysis. Generalizability to chronic and non-traumatic DoC requires dedicated studies.	Wu et al. (2023), Yang et al. (2025), and Wang et al. (2022)
taVNS	Frequency, intensity, and pulse width	20–25 Hz; 0.1–8 mA; 100–500 μs pulse width	Limited etiology-specific data; mixed cohorts studied	MCS responds at 4 weeks; UWS: no significant short-term effect but 6-month case shows delayed improvement	Substantial parameter heterogeneity limits cross-study comparison. Fixed intensity used in DoC (perception threshold calibration infeasible). MCS patients show behavioral response; UWS response limited to fNIRS connectivity changes. Optimal parameters undetermined.	Zhou et al. (2023), Gao et al. (2025), Osińska et al. (2022), and Zou et al. (2024)
tFUS	Acoustic frequency, PRF, intensity, and target	250–650 kHz; PRF 100 Hz; ISPPA 720 mW/cm^2^; thalamic target	Studied in mixed etiologies (acute and chronic); too few patients for subgroup analysis	Acute DoC: 4/11 improved after single session; chronic DoC: 4/10 improved after 2 sessions	Unique capacity to target thalamus non-invasively. Directly addresses mesocircuit bottleneck. Extremely small samples (*n =* 1–11); no sham controls. Optimal parameters (frequency, PRF, intensity) completely unknown. Rapid translation from bench to bedside is notable.	Monti et al. (2016), Cain et al. (2022), and Cain et al. (2021)
SCS	Stimulation frequency	5 Hz vs. 70 Hz; cervical epidural; 2 weeks	TBI and traumatic etiology: better prognosis; brainstem hemorrhage: >70% respond	MCS− > MCS+ > UWS; 70 Hz effects persist 1 week post-treatment	Both 5 Hz and 70 Hz promote consciousness recovery, but 70 Hz shows greater and more sustained CRS-R improvement. MCS − shows greatest benefit. EEG ABCD model enables preoperative responder prediction. Frequency optimization and closed-loop approaches needed.	Zhuang et al. (2022), and Zhuang et al. (2024), Huang et al. (2023b), Sun et al. (2024), Yang et al. (2022), and Piedade et al. (2023)

rTMS, repetitive transcranial magnetic stimulation; tDCS, transcranial direct current stimulation; HD-tDCS, high-definition tDCS; MNS, median nerve stimulation; taVNS, transcutaneous auricular vagus nerve stimulation; tFUS, transcranial focused ultrasound; SCS, spinal cord stimulation; DLPFC, dorsolateral prefrontal cortex; M1, primary motor cortex; RMT, resting motor threshold; PRF, pulse repetition frequency; ISPPA, spatial-peak pulse-average intensity; CRS-R, Coma Recovery Scale-Revised; GCS, Glasgow Coma Scale; TBI, traumatic brain injury; MCS, minimally conscious state; UWS, unresponsive wakefulness syndrome; ARAS, ascending reticular activating system; NS, not significant; EEG, electroencephalography; fNIRS, functional near-infrared spectroscopy; DoC, disorders of consciousness; RR, risk ratio; VS, vegetative state; mA, milliampere; μs, microsecond.

The integration of neuroimaging and electrophysiological biomarkers into (He et al., 2020) neuromodulation research into precision medicine. EEG-derived markers have shown considerable promise: higher baseline alpha power and lower delta power predict favorable rTMS response, theta-band network centrality correlates with tDCS responsiveness (Thibaut et al., 2018), and the PCI can quantify the (Xu et al., 2024). Resting-state fMRI connectivity within the default mode network and frontoparietal network has been associated with recovery trajectories and may serve as prognostic biomarkers (Wang et al., 2024; Kazazian et al., 2025). A recent prospective study demonstrated that intact default mode network connectivity in acute TBI patients was associated with better six-month functional outcomes, even in behaviorally unresponsive individuals (Bodien et al., 2025b). Critically, these biomarkers could move beyond diagnostic utility to actively guide therapeutic decision-making—for example, patients with preserved frontoparietal alpha connectivity might be preferentially triaged to DLPFC-targeted rTMS protocols, while those with predominant thalamocortical disconnection might benefit more from tFUS or SCS directed at subcortical targets. It is important to emphasize, however, that alpha activity, P300 responses, and PCI values currently remain promising research tools rather than clinically validated predictive biomarkers. None of these markers has yet been prospectively validated in adequately powered trials for routine clinical stratification of treatment candidacy, and their incorporation into clinical decision-making should be considered exploratory pending further validation. Furthermore, stratification by chronicity is essential, as the neuroplastic window may narrow over time; a dose–response relationship between treatment duration and efficacy has been confirmed for MNS (Wang et al., 2022), and the prognostic advantage of early intervention (≤7 days) has been demonstrated for HBOT (Li S. et al., 2024). Etiology-specific considerations are equally important, with traumatic DoC patients consistently showing better neuromodulation responses compared to anoxic or vascular etiologies across multiple modalities (Liu et al., 2025; Yang et al., 2022; Thibaut et al., 2023), possibly reflecting differences in the extent of diffuse axonal injury versus gray matter necrosis and their differential impact on mesocircuit integrity.

The neuroplasticity mechanisms underlying consciousness recovery through neuromodulation can be further illuminated by drawing parallels to network reorganization observed in other severe neurological conditions. In subcortical stroke, Fan et al. (2026) demonstrated that dynamic interhemispheric interactions reveal compensatory pathways: specifically, during a segregation state characterized by reduced homotopic dorsal premotor cortex synchronization, the contralesional premotor cortex establishes strengthened functional connectivity with bilateral subcortical networks, effectively compensating for impaired ipsilesional motor cortex function. This state-dependent compensatory mechanism—whereby the unaffected hemisphere assumes functions of the damaged hemisphere through enhanced subcortical interactions—bears direct relevance to DoC, where neuromodulation techniques such as rTMS targeting the DLPFC or angular gyrus may similarly recruit preserved contralateral or posterior cortical networks to compensate for focal damage. Moreover, Wu et al. (2025) have shown that structural brain asymmetry is dynamically shaped by synaptic pruning, corpus callosum integrity, and environmental factors, with asymmetric developmental trajectories linked to both cognitive capacity and vulnerability to neuropsychiatric conditions. These findings, though derived from developmental cohorts, underscore a fundamental principle applicable to DoC: that large-scale brain network asymmetry and interhemispheric balance are not static properties but dynamic states amenable to modulation. In the context of severe brain injury, disrupted hemispheric balance may be partially restored through neuromodulation, particularly when treatment protocols are informed by individual patterns of hemispheric connectivity assessed through resting-state fMRI or TMS-EEG (Wu et al., 2025; Gao Y. et al., 2025).

The emerging evidence for combined interventions—such as rTMS plus MNS (Xiong et al., 2023), tDCS plus median nerve stimulation (Chen et al., 2025), HBOT with simultaneous MNS (Liu et al., 2022), and the proposed taVNS plus HD-tDCS protocols (Zhuang et al., 2023)—demands critical evaluation of the potential neurobiological mechanisms and overlapping neural pathways that may underlie their synergistic effects, rather than merely documenting behavioral superiority over single-modality approaches. From the mesocircuit perspective, the rationale for combination therapies lies in simultaneously engaging multiple nodes of the disrupted circuit: cortical stimulation (rTMS/tDCS) directly enhances frontoparietal excitability and top-down processing, while peripheral nerve stimulation (MNS/taVNS) provides bottom-up afferent drive through the ascending reticular activating system and locus coeruleus-norepinephrine pathways, and SCS activates the reticulo-thalamo-cortical pathway to increase cerebral blood flow (Piedade et al., 2023). The superior efficacy of simultaneous HBOT-MNS over sequential administration (Liu et al., 2022) further suggests that temporal coupling of these complementary mechanisms—enhanced oxygen delivery coinciding with increased neural activation—may optimize conditions for synaptic plasticity. At the molecular level, convergent effects on dopaminergic neurotransmission (implicated in both MNS-mediated miRNA changes (Jia et al., 2022) and tFUS-induced striatal dopamine release (Olaitan et al., 2025)), BDNF-dependent neuroplasticity, and GABAergic/glutamatergic balance modulation (Galanis et al., 2025) may explain why multimodal approaches exceed additive benefits. However, these mechanistic interpretations remain largely hypothetical and are extrapolated from preclinical models; direct human evidence for the proposed synergistic pathways is limited, highlighting the need for mechanistic studies using concurrent neuroimaging during combined interventions.

Perhaps the most critical gap in the current evidence base concerns the long-term durability of neuromodulatory effects and whether transient neurophysiological changes reliably translate into meaningful functional independence or sustained quality-of-life improvements. Most studies in this review report outcomes only at the end of the intervention period or at short-term follow-up (typically 1–4 weeks), with few exceptions: the 6-month follow-up data from Yang et al. for SCS (Yang et al., 2022), the 3-month tDCS data from Thibaut et al. (2023), and the 6-month MNS recovery data from Wu et al. (2023). Sierra et al. (2025) recently demonstrated that individuals with prolonged DoC who emerged from the minimally conscious state generally showed limited functional recovery during the first year, with severe disability persisting despite behavioral improvement in consciousness levels. This disconnect between consciousness recovery (as measured by CRS-R) and functional independence underscores the need for trials to incorporate patient-centered outcome measures beyond the CRS-R, including the Disability Rating Scale, functional independence metrics, and quality-of-life assessments. The question of whether neurophysiological gains induced by neuromodulation—such as enhanced EEG alpha power, improved frontoparietal connectivity, or increased perturbational complexity—represent durable neuroplastic reorganization or merely transient state changes is fundamental to (Gao Y. et al., 2025) with extended follow-up periods (6–12 months minimum), coupled with serial neuroimaging to track evolving connectivity patterns, are essential to bridge this translational gap. Furthermore, the development of adaptive closed-loop protocols—in which stimulation parameters are continuously adjusted based on real-time EEG or fMRI biomarkers of brain state—represents a promising strategy for maintaining therapeutic effects and transitioning from fixed, population-level stimulation paradigms to truly individualized treatment approaches (Gao Y. et al., 2025; Wan et al., 2024b).

## 2 Conclusion

Neuromodulation techniques may represent a promising therapeutic avenue for DoC. Among the reviewed approaches high-frequency rTMS (10–20 Hz) targeting the DLPFC, right median nerve stimulation, and short-term spinal cord stimulation currently appear to have comparatively stronger support. Right MNS demonstrated significant consciousness recovery rates in acute traumatic coma (RR = 1.36, 95% CI 1.18–1.56), while rTMS has shown sustained CRS-R improvements and enhanced cortical connectivity. Among these, right MNS has accumulated the most robust RCT-level and meta-analytic evidence (Level 1b), while high-frequency rTMS is supported by multiple RCTs with moderate-to-large samples (Level 2a). Other modalities, including tFUS, tPBM, and tACS, remain at the preliminary or exploratory stage (Level 3b–4) and require further validation before definitive clinical conclusions can be drawn. Importantly, the current evidence base is limited by predominantly short-term outcomes, small sample sizes, and heterogeneous stimulation protocols, underscoring the need for caution in interpreting therapeutic claims.

Treatment responses appear to vary by consciousness level (MCS superior to UWS), etiology (traumatic better than non-traumatic), and individual factors. Mechanistically, techniques converge on restoring frontoparietal connectivity, modulating thalamocortical circuits, and enhancing synaptic plasticity. EEG biomarkers show promise for predicting treatment response. These findings can be integrated within the mesocircuit hypothesis framework, which posits that anterior forebrain dysfunction following severe brain injury results from disrupted thalamocortical and thalamostriatal excitatory drive. Different neuromodulation modalities appear to target overlapping elements of these disrupted circuits, though the precise mechanisms remain to be fully elucidated, as many mechanistic interpretations currently derive from preclinical models rather than direct human evidence. It is also important to note that the long-term durability of neuromodulatory effects and their translation into meaningful functional independence remain insufficiently characterized in the existing literature.

Future priorities include large-scale multicenter RCTs, predictive biomarker development, closed-loop stimulation paradigms, and systematic investigation of combination strategies. The field would benefit from transitioning to individualized, probabilistic frameworks. Safety profiles have generally appeared favorable across modalities. While emerging evidence suggests potential therapeutic value of neuromodulation for DoC, definitive conclusions regarding clinical efficacy await confirmation from adequately powered, rigorously designed clinical trials with long-term functional outcome measures and patient-centered endpoints.

## 3 Limitations

The current evidence base has several important limitations. Most studies involve small sample sizes (typically 10–40 patients) and employ heterogeneous protocols, hindering definitive conclusions and optimal parameter identification. Study designs vary in rigor, with many lacking adequate blinding or sham controls. Outcome measures are inconsistent across studies, and long-term functional outcomes remain insufficiently characterized. Geographic concentration of certain studies (particularly for acupuncture) raises concerns about publication bias. Mechanistic understanding of consciousness recovery and reliable predictors of treatment response remain incomplete. This narrative review provides qualitative synthesis rather than quantitative meta-analysis and may not have captured all relevant studies, particularly non-English publications. As this is a narrative review without a formal PRISMA workflow or risk-of-bias assessment, caution should be exercised when interpreting statements about the overall strength of evidence across modalities.

## Glossary

ABCD: amplitude-frequency-complexity-directionality (EEG model)
Ang-2: angiopoietin-2
ARAS: ascending reticular activating system
ATA: absolute atmospheres
BDNF: brain-derived neurotrophic factor
BOLD: blood oxygen level-dependent
CI: confidence interval
CNS: central nervous system
CNKI: China National Knowledge Infrastructure
CRS-R: Coma Recovery Scale-Revised
DLPFC: dorsolateral prefrontal cortex
DoC: disorders of consciousness
DRN: dorsal raphe nucleus
EEG: electroencephalography
fMRI: functional magnetic resonance imaging
fNIRS: functional near-infrared spectroscopy
FPN: frontoparietal network
FOUR: Full Outline of UnResponsiveness
GCS: Glasgow Coma Scale
GOSE: Glasgow Outcome Scale-Extended
HBOT: hyperbaric oxygen therapy
HD-tDCS: high-definition transcranial direct current stimulation
HRV: heart rate variability
HRV-HF: heart rate variability high frequency
IP-10: interferon gamma-induced protein 10
ISPPA: spatial-peak pulse-average intensity
iTBS: intermittent theta burst stimulation
ICU: intensive care unit
LC: locus coeruleus
LF: low frequency
LIFU: low-intensity focused ultrasound
M1: primary motor cortex
MCS: minimally conscious state
MD: mean difference
MNES: median nerve electrical stimulation
MNS: median nerve stimulation
MRI: magnetic resonance imaging
MSN: medium spiny neuron
N20: somatosensory evoked potential N20 component
N200: event-related potential N200 component
NIBS: non-invasive brain stimulation
NIR: near-infrared
NTS: nucleus tractus solitarius
OCEBM: Oxford Centre for Evidence-Based Medicine
OR: odds ratio
P300: event-related potential P300 component
pDoC: prolonged disorders of consciousness
PCI: Perturbational Complexity Index
PRF: pulse repetition frequency
PVS: persistent vegetative state
RCT: randomized controlled trial
RMNS: right median nerve stimulation
RMT: resting motor threshold
RR: risk ratio
rTMS: repetitive transcranial magnetic stimulation
SampEn: sample entropy
SCS: spinal cord stimulation
SEP: somatosensory evoked potential
SR: systematic review
st-SCS: short-term spinal cord stimulation
tACS: transcranial alternating current stimulation
taVNS: transcutaneous auricular vagus nerve stimulation
TBI: traumatic brain injury
TBS: theta burst stimulation
tDCS: transcranial direct current stimulation
tES: transcranial electrical stimulation
tFUS: transcranial focused ultrasound
TMS: transcranial magnetic stimulation
TMS-EEG: transcranial magnetic stimulation combined with electroencephalography
TNFα: tumor necrosis factor alpha
tPBM: transcranial photobiomodulation
TRAF3: TNF receptor-associated factor 3
TRAAK: TWIK-related arachidonic acid-stimulated K^+^ channel
tRNS: transcranial random noise stimulation
TCM: traditional Chinese medicine
UWS: unresponsive wakefulness syndrome
VEGF: vascular endothelial growth factor
VEGF-C: vascular endothelial growth factor C
VLF: very low frequency
VNS: vagus nerve stimulation
VS: vegetative state.

## References

[ref1] Aberra A. S. Wang R. Grill W. M. Peterchev A. V. (2023). Multi-scale model of axonal and dendritic polarization by transcranial direct current stimulation in realistic head geometry. Brain Stimul. 16, 1776–1791. doi: 10.1016/j.brs.2023.11.018, 38056825 PMC10842743

[ref2] Allison T. McCarthy G. Wood C. C. Jones S. J. (1991). Potentials evoked in human and monkey cerebral cortex by stimulation of the median nerve. A review of scalp and intracranial recordings. Brain 114, 2465–2503. doi: 10.1093/brain/114.6.2465 1782527

[ref3] Aloi D. della Rocchetta A. I. Ditchfield A. Coulborn S. Fernández-Espejo D. (2021). Therapeutic use of transcranial direct current stimulation in the rehabilitation of prolonged disorders of consciousness. Front. Neurol. 12:632572. doi: 10.3389/fneur.2021.632572, 33897592 PMC8058460

[ref4] Aloi D. Jalali R. Calzolari S. Lafanechere M. Miall R. C. Fernández-Espejo D. (2023). Multi-session tDCS paired with passive mobilisation of the thumb modulates thalamo-cortical coupling during command following in the healthy brain. NeuroImage 274:120145. doi: 10.1016/j.neuroimage.2023.120145, 37121374

[ref5] Aloi D. Jalali R. Tilsley P. Miall R. C. Fernández-Espejo D. (2022). tDCS modulates effective connectivity during motor command following; a potential therapeutic target for disorders of consciousness. NeuroImage 247:118781. doi: 10.1016/j.neuroimage.2021.118781, 34879252 PMC8803542

[ref6] Anderson M. C. Crespo-Garcia M. Subbulakshmi S. (2025). Brain mechanisms underlying the inhibitory control of thought. Nat. Rev. Neurosci. 26, 415–437. doi: 10.1038/s41583-025-00929-y, 40379896

[ref7] Angerer M. Pichler G. Angerer B. Scarpatetti M. Schabus M. Blume C. (2022). From dawn to dusk-mimicking natural daylight exposure improves circadian rhythm entrainment in patients with severe brain injury. Sleep 45:065. doi: 10.1093/sleep/zsac065, 35290446 PMC9272242

[ref8] Antoniou A. Evripidou N. Damianou C. (2024). Focused ultrasound heating in brain tissue/skull phantoms with 1 MHz single-element transducer. J. Ultrasound 27, 263–274. doi: 10.1007/s40477-023-00810-7, 37517052 PMC11178743

[ref9] Avalos-Alais S. Jedynak M. Boyer A. Chanteloup-Forêt B. Pinheiro C. Cline C. C. . (2026). High-resolution electrophysiological mapping of effective connectivity of lateral prefrontal cortex. Brain 149, 963–975. doi: 10.1093/brain/awaf317, 40924875 PMC13016832

[ref10] Bai Y. Yang L. Meng X. Huang Y. Wang Q. Gong A. . (2024). Breakdown of effective information flow in disorders of consciousness: Insights from TMS-EEG. Brain Stimul. 17, 533–542. doi: 10.1016/j.brs.2024.04.011 38641169

[ref11] Barone A. Senerchia G. de Simone G. Manzo M. Ciccarelli M. Tozza S. . (2025). In vivo assessment of GABAergic inhibition and glutamate facilitation in treatment-resistant schizophrenia: a TMS study integrating clinical, cognitive, and neurophysiological evaluations. Schizophrenia 11:90. doi: 10.1038/s41537-025-00634-w, 40537471 PMC12179267

[ref12] Barra M. E. Solt K. Yu X. Edlow B. L. (2024). Restoring consciousness with pharmacologic therapy: mechanisms, targets, and future directions. Neurotherapeutics 21:e00374. doi: 10.1016/j.neurot.2024.e00374, 39019729 PMC11452330

[ref13] Bartolini D. Liuzzi P. Secci S. Hakiki B. Scarpino M. Burali R. . (2025). Temporal network instability and low-frequency overconnectivity underlie disorders of consciousness in severe brain injury. Sci. Rep. 15:38835. doi: 10.1038/s41598-025-09233-2, 41198756 PMC12592492

[ref14] Benussi A. Cantoni V. Rivolta J. Zoppi N. Cotelli M. S. Bianchi M. . (2024). Alpha tACS improves cognition and modulates neurotransmission in dementia with Lewy bodies. Mov. Disord. 39, 1993–2003. doi: 10.1002/mds.29969 39136447

[ref15] Bertoni T. Noel J. P. Bockbrader M. Foglia C. Colachis S. Orset B. . (2025). Pre-movement sensorimotor oscillations shape the sense of agency by gating cortical connectivity. Nat. Commun. 16:3594. doi: 10.1038/s41467-025-58683-9, 40234393 PMC12000325

[ref16] Bhattacharya A. Darmani G. Udupa K. Nankoo J. F. Ding M. Y. R. Chen R. (2025). Induction of plasticity and metaplasticity using noninvasive brain stimulation. Trends Neurosci. 48, 792–807. doi: 10.1016/j.tins.2025.07.009 40813159

[ref17] Bin-Alamer O. Abou-al-Shaar H. Efrati S. Hadanny A. Beckman R. L. Elamir M. . (2024). Hyperbaric oxygen therapy as a neuromodulatory technique: a review of the recent evidence. Front. Neurol. 15:1450134. doi: 10.3389/fneur.2024.1450134, 39445195 PMC11496187

[ref18] Block N. (2024). What does decoding from the PFC reveal about consciousness? Trends Cogn. Sci. 28, 804–813. doi: 10.1016/j.tics.2024.05.004 38862352

[ref19] Bodien Y. G. Allanson J. Cardone P. Bonhomme A. Carmona J. Chatelle C. . (2024). Cognitive motor dissociation in disorders of consciousness. N. Engl. J. Med. 391, 598–608. doi: 10.1056/NEJMoa2400645, 39141852 PMC7617195

[ref20] Bodien Y. G. Busl K. M. Chang C. W. J. Claassen J. Gaspard N. Gosseries O. . (2025a). Disorders of consciousness diagnosis, interventions, and prognostication for the intensivist: report of the 2025 ISICEM roundtable. Intensive Care Med. 52, 42–62. doi: 10.1007/s00134-025-08224-1 41396553 PMC12852174

[ref21] Bodien Y. G. Fecchio M. Gilmore N. Freeman H. J. Sanders W. R. Meydan A. . (2025b). Acute biomarkers of consciousness are associated with recovery after severe traumatic brain injury. J. Neurotrauma 43. doi: 10.1177/08977151251377469 PMC13191150 41182259

[ref22] Booth S. J. Taylor J. R. Brown L. J. E. Pobric G. (2022). The effects of transcranial alternating current stimulation on memory performance in healthy adults: a systematic review. Cortex 147, 112–139. doi: 10.1016/j.cortex.2021.12.001, 35032750

[ref23] Cain J. A. Spivak N. M. Coetzee J. P. Crone J. S. Johnson M. A. Lutkenhoff E. S. . (2021). Ultrasonic thalamic stimulation in chronic disorders of consciousness. Brain Stimul. 14, 301–303. doi: 10.1016/j.brs.2021.01.008, 33465497

[ref24] Cain J. A. Spivak N. M. Coetzee J. P. Crone J. S. Johnson M. A. Lutkenhoff E. S. . (2022). Ultrasonic deep brain neuromodulation in acute disorders of consciousness: a proof-of-concept. Brain Sci. 12:428. doi: 10.3390/brainsci12040428, 35447960 PMC9032970

[ref25] Carrière M. Mortaheb S. Raimondo F. Annen J. Barra A. Binda Fossati M. C. . (2020). Neurophysiological correlates of a single session of prefrontal tDCS in patients with prolonged disorders of consciousness: a pilot double-blind randomized controlled study. Brain Sci. 10:469. doi: 10.3390/brainsci10070469, 32708119 PMC7408434

[ref26] Chang Q. Y. Lin Y. W. Hsieh C. L. (2018). Acupuncture and neuroregeneration in ischemic stroke. Neural Regen. Res. 13, 573–583. doi: 10.4103/1673-5374.230272, 29722298 PMC5950656

[ref27] Chau B. K. H. Law C. K. To J. Y. L. Shum D. H. K. Mars R. B. (2025). Complex functions of human lateral frontopolar cortex. Brain 148, 3833–3843. doi: 10.1093/brain/awaf289 40795209 PMC12588682

[ref28] Chen R. Gao Q. Wu D. W. Hao J. Zhao J. J. Wang X. . (2025). Effect of cerebellar stimulation on awareness recovery in disorders of consciousness (CARE-DoC): a randomized, sham-controlled, crossover clinical trial. Neurotherapeutics 22:e00635. doi: 10.1016/j.neurot.2025.e00635, 40617737 PMC12491794

[ref29] Chen C. H. Hsieh C. L. (2020). Effect of acupuncture on oxidative stress induced by cerebral ischemia-reperfusion injury. Antioxidants 9:248. doi: 10.3390/antiox9030248 32204376 PMC7139408

[ref30] Chen H. Huang S. Chen S. Wang Z. Luo Y. Guo Y. . (2025). Impact of transcranial direct current stimulation combined with median nerve stimulation on CRS-R in patients with prolonged disorders of consciousness after cerebral hemorrhage: protocol for a randomized controlled trial. Front. Neurol. 16:1497316. doi: 10.3389/fneur.2025.1497316, 40556649 PMC12185530

[ref31] Chen Y. Lu X. Hu L. (2023). Transcutaneous auricular Vagus nerve stimulation facilitates cortical arousal and alertness. Int. J. Environ. Res. Public Health 20:1402. doi: 10.3390/ijerph20021402 36674156 PMC9859411

[ref32] Cincotta M. Giovannelli F. Chiaramonti R. Bianco G. Godone M. Battista D. . (2015). No effects of 20 Hz-rTMS of the primary motor cortex in vegetative state: a randomised, sham-controlled study. Cortex 71, 368–376. doi: 10.1016/j.cortex.2015.07.027 26301875

[ref33] Comanducci A. Boly M. Claassen J. de Lucia M. Gibson R. M. Juan E. . (2020). Clinical and advanced neurophysiology in the prognostic and diagnostic evaluation of disorders of consciousness: review of an IFCN-endorsed expert group. Clin. Neurophysiol. 131, 2736–2765. doi: 10.1016/j.clinph.2020.07.015 32917521

[ref34] Cooper J. B. Jane J. O. H. N. A. Alves W. A. Y. N. E. M. Cooper E. D. W. I. N. B. (1999). Right median nerve electrical stimulation to hasten awakening from coma. Brain Inj. 13, 261–267. doi: 10.1080/026990599121638, 10230527

[ref35] Courties A. Tuffet S. Cormier G. Roux C. H. Ornetti P. Pers Y. M. . (2025). Transcutaneous auricular vagus nerve stimulation versus sham stimulation in patients with erosive hand osteoarthritis (ESTIVAL): a randomised, multicentre, double-blind, sham-controlled trial. Lancet. Rheumatol 8, e98–e107. doi: 10.1016/S2665-9913(25)00226-7 41380709

[ref36] Crone J. S. Schurz M. Höller Y. Bergmann J. Monti M. Schmid E. . (2015). Impaired consciousness is linked to changes in effective connectivity of the posterior cingulate cortex within the default mode network. NeuroImage 110, 101–109. doi: 10.1016/j.neuroimage.2015.01.037, 25620493 PMC4389831

[ref37] Cruse D. Thibaut A. Demertzi A. Nantes J. C. Bruno M. A. Gosseries O. . (2013). Actigraphy assessments of circadian sleep-wake cycles in the vegetative and minimally conscious states. BMC Med. 11:18. doi: 10.1186/1741-7015-11-18 23347467 PMC3606428

[ref38] Darrow D. P. (2019). Focused ultrasound for neuromodulation. Neurotherapeutics 16, 88–99. doi: 10.1007/s13311-018-00691-3, 30488340 PMC6361056

[ref39] Davidson B. Schmidt F. A. Bichsel O. Hajiabadi M. M. Lozano A. M. (2025). Transcranial focused ultrasound: a transformative tool for intracranial ablation, drug delivery, and neuromodulation. IEEE Rev Biomed. Eng. 19, 201–215. doi: 10.1109/RBME.2025.3624970 41217925

[ref40] Day A. S. Lue J. H. Sun W. Z. Shieh J. Y. Wen C. Y. (2001). A beta-fiber intensity stimulation of chronically constricted median nerve induces c-fos expression in thalamic projection neurons of the cuneate nucleus in rats with behavioral signs of neuropathic pain. Brain Res. 895, 194–203. doi: 10.1016/S0006-8993(01)02095-9 11259778

[ref41] Demertzi A. Antonopoulos G. Heine L. Voss H. U. Crone J. S. de Los Angeles C. . (2015). Intrinsic functional connectivity differentiates minimally conscious from unresponsive patients. Brain 138, 2619–2631. doi: 10.1093/brain/awv169, 26117367

[ref42] Dong L. Li H. Dang H. Zhang X. Yue S. Zhang H. (2023). Efficacy of non-invasive brain stimulation for disorders of consciousness: a systematic review and meta-analysis. Front. Neurosci. 17:1219043. doi: 10.3389/fnins.2023.1219043, 37496734 PMC10366382

[ref43] Durand N. C. Kim H. G. Patel V. N. Turnbull M. T. Siegel J. L. Hodge D. O. . (2024). Mesenchymal stem cell therapy in acute intracerebral hemorrhage: a dose-escalation safety and tolerability trial. Neurocrit. Care. 41, 59–69. doi: 10.1007/s12028-023-01897-w, 38114796 PMC11335835

[ref44] Dutta R. R. Abdolmanafi S. Rabizadeh A. Baghbaninogourani R. Mansooridara S. Lopez A. . (2025). Neuromodulation and disorders of consciousness: systematic review and pathophysiology. Neuromodulation 28, 380–400. doi: 10.1016/j.neurom.2024.09.003, 39425733

[ref45] Edlow B. L. Claassen J. Schiff N. D. Greer D. M. (2021). Recovery from disorders of consciousness: mechanisms, prognosis and emerging therapies. Nat. Rev. Neurol. 17, 135–156. doi: 10.1038/s41582-020-00428-x, 33318675 PMC7734616

[ref46] Fan H. Wang H. Lian Z. Yu Q. Wu X. Kuang N. . (2026). Dynamic interactions between hemispheres reveal a compensatory pathway for motor recovery in moderate-to-severe subcortical stroke. J Stroke 28, 97–114. doi: 10.5853/jos.2025.01725, 41478717 PMC12883883

[ref47] Fan J. Zhong Y. Wang H. Aierken N. He R. (2022). Repetitive transcranial magnetic stimulation improves consciousness in some patients with disorders of consciousness. Clin. Rehabil. 36, 916–925. doi: 10.1177/02692155221089455 35322709

[ref48] Farisco M. Evers K. Annen J. Blandin V. Camassa A. Cecconi B. . (2026). Advancing the science of consciousness: from ethics to clinical care. Neurosci. Biobehav. Rev. 180:106497. doi: 10.1016/j.neubiorev.2025.106497, 41319843

[ref49] Fathi Y. Dissassuca F. Ricci G. Liberati G. (2025). Individualised alpha-tACS for modulating pain perception and neural oscillations: a sham-controlled study in healthy participants. Eur. J. Pain 29:e70086. doi: 10.1002/ejp.70086 40682416

[ref50] Feng Z. Du Q. (2016). Mechanisms responsible for the effect of median nerve electrical stimulation on traumatic brain injury-induced coma: orexin-A-mediated N-methyl-D-aspartate receptor subunit NR1 upregulation. Neural Regen. Res. 11, 951–956. doi: 10.4103/1673-5374.184494, 27482224 PMC4962593

[ref51] Fransson P. Marrelec G. (2008). The precuneus/posterior cingulate cortex plays a pivotal role in the default mode network: evidence from a partial correlation network analysis. NeuroImage 42, 1178–1184. doi: 10.1016/j.neuroimage.2008.05.059, 18598773

[ref52] Galanis C. Hananeia N. Lenz M. Vasheghani Farahani M. Jedlicka P. Vlachos A. (2025). Repetitive magnetic stimulation induces plasticity of excitatory synapses through cooperative pre- and postsynaptic activity. Brain Stimul. 18, 1641–1650. doi: 10.1016/j.brs.2025.08.019 40850522

[ref53] Gangemi A. de Luca R. Fabio R. A. Bonanno M. Cardile D. Mignacca M. R. . (2024). Cognitive effects of transcranial direct current stimulation plus robotic Verticalization in minimally conscious state. Biomedicine 12:244. doi: 10.3390/biomedicines12102244, 39457557 PMC11504468

[ref54] Gangemi A. Impellizzeri F. Fabio R. A. Suriano R. D’Arrigo A. Rifici C. . (2025). Cognitive and neurophysiological effects of bilateral tDCS neuromodulation in patients with minimally conscious state. Sci. Rep. 15:14389. doi: 10.1038/s41598-025-99591-8 40274956 PMC12022346

[ref55] Gao Y. Bai J. Gu Z. Xie Y. An W. Liu Z. . (2025). Noninvasive neuromodulation for disorders of consciousness: an updated systematic review and meta-analysis. Crit. Care 29:269. doi: 10.1186/s13054-025-05429-0 40611258 PMC12224763

[ref56] Gao Q. Hao J. Kang X. Yuan F. Liu Y. Chen R. . (2023). EEG dynamics induced by zolpidem forecast consciousness evolution in prolonged disorders of consciousness. Clin. Neurophysiol. 153, 46–56. doi: 10.1016/j.clinph.2023.06.012 37454563

[ref57] Gao F. Wang L. Wang Z. Tian Y. Wu J. Wang M. . (2025). Case report: monitoring consciousness with fNIRS in a patient with prolonged reduced consciousness following hemorrhagic stroke undergoing adjunct taVNS therapy. Front. Neurosci. 19:1519939. doi: 10.3389/fnins.2025.1519939, 39967804 PMC11832507

[ref58] Ge X. Zhang Y. Xin T. Luan X. (2021). Effects of 10 Hz repetitive transcranial magnetic stimulation of the right dorsolateral prefrontal cortex in the vegetative state. Exp. Ther. Med. 21:206. doi: 10.3892/etm.2021.9626, 33500699 PMC7818534

[ref59] Gerges A. N. H. Graetz L. Hillier S. Uy J. Hamilton T. Opie G. . (2025). Transcutaneous auricular vagus nerve stimulation modifies cortical excitability in middle-aged and older adults. Psychophysiology 62:e14584. doi: 10.1111/psyp.14584, 38602055 PMC11780349

[ref60] Giacino J. T. Ashwal S. Childs N. Cranford R. Jennett B. Katz D. I. . (2002). The minimally conscious state: definition and diagnostic criteria. Neurology 58, 349–353. doi: 10.1212/WNL.58.3.349 11839831

[ref61] Giacino J. T. Fins J. J. Laureys S. Schiff N. D. (2014). Disorders of consciousness after acquired brain injury: the state of the science. Nat. Rev. Neurol. 10, 99–114. doi: 10.1038/nrneurol.2013.279 24468878

[ref62] Giraudier M. Ventura-Bort C. Weymar M. (2024). Effects of transcutaneous auricular Vagus nerve stimulation on the P300: do stimulation duration and stimulation type matter? Brain Sci. 14:690. doi: 10.3390/brainsci14070690, 39061430 PMC11274684

[ref63] Gong Y. Zhang L. Zhang Y. Cao J. Wang Y. T. Lei Y. . (2025). Repetitive transcranial magnetic stimulation targeting the dorsolateral prefrontal cortex promotes recovery of consciousness in patients with disorders of consciousness: a meta-analysis of randomized controlled trials. J. Neurol. 272:699. doi: 10.1007/s00415-025-13437-x, 41094293

[ref64] Gooley J. J. Chamberlain K. Smith K. A. Khalsa S. B. S. Rajaratnam S. M. W. van Reen E. . (2011). Exposure to room light before bedtime suppresses melatonin onset and shortens melatonin duration in humans. J. Clin. Endocrinol. Metab. 96, E463–E472. doi: 10.1210/jc.2010-2098 21193540 PMC3047226

[ref65] Guo Y. Bai Y. Xia X. Li J. Wang X. Dai Y. . (2019). Effects of Long-lasting high-definition transcranial direct current stimulation in chronic disorders of consciousness: a pilot study. Front. Neurosci. 13:412. doi: 10.3389/fnins.2019.00412, 31114475 PMC6502996

[ref66] Guo X. Zhang X. Sun M. Yu L. Qian C. Zhang J. . (2022). Modulation of brain rhythm oscillations by Xingnao Kaiqiao acupuncture correlates with stroke recovery: a randomized control trial. J Integr Complement Med 28, 436–444. doi: 10.1089/jicm.2021.0264 35275751

[ref67] Gurtubay I. G. Perez-Rodriguez D. R. Fernandez E. Librero-Lopez J. Calvo D. Bermejo P. . (2023). Immediate effects and duration of a short and single application of transcutaneous auricular vagus nerve stimulation on P300 event related potential. Front. Neurosci. 17:1096865. doi: 10.3389/fnins.2023.1096865, 37051148 PMC10083261

[ref68] Hamblin M. R. (2016). Shining light on the head: Photobiomodulation for brain disorders. BBA Clin 6, 113–124. doi: 10.1016/j.bbacli.2016.09.002, 27752476 PMC5066074

[ref199] He H. Sun X. Doose J. Faller J. McIntosh J. R. Saber G. T. . (2025). TMS-induced modulation of brain networks and its associations to rTMS treatment for depression: a concurrent fMRI-EEG-TMS study. Brain Stimul. 18, 1955–1965. doi: 10.1016/j.brs.2025.10.013 41109521 PMC12716231

[ref69] He R. Fan J. Wang H. Zhong Y. Ma J. (2020). Differentiating responders and non-responders to rTMS treatment for disorder of consciousness using EEG after-effects. Front. Neurol. 11:583268. doi: 10.3389/fneur.2020.583268 33329325 PMC7714935

[ref70] He R. H. Wang H. J. Zhou Z. Fan J. Z. Zhang S. Q. Zhong Y. H. (2021). The influence of high-frequency repetitive transcranial magnetic stimulation on endogenous estrogen in patients with disorders of consciousness. Brain Stimul. 14, 461–466. doi: 10.1016/j.brs.2021.02.014, 33677157

[ref71] He F. Wu M. Meng F. Hu Y. Gao J. Chen Z. . (2018). Effects of 20 Hz repetitive transcranial magnetic stimulation on disorders of consciousness: a resting-state electroencephalography study. Neural Plast. 2018:5036184. doi: 10.1155/2018/5036184 29770146 PMC5889874

[ref72] Hermann B. Raimondo F. Hirsch L. Huang Y. Denis-Valente M. Pérez P. . (2020). Combined behavioral and electrophysiological evidence for a direct cortical effect of prefrontal tDCS on disorders of consciousness. Sci. Rep. 10:4323. doi: 10.1038/s41598-020-61180-2, 32152347 PMC7062738

[ref73] Hilz M. J. (2022). Transcutaneous vagus nerve stimulation - a brief introduction and overview. Auton. Neurosci. 243:103038. doi: 10.1016/j.autneu.2022.103038, 36201901

[ref74] Ho H. C. Chen W. S. Hsiao M. Y. (2025). Neuromodulation effects of low-intensity transcranial focused ultrasound in human, a systematic review focusing on motor and sensory functions. J. Neuroeng. Rehabil. 22:254. doi: 10.1186/s12984-025-01722-9, 41316352 PMC12664190

[ref75] Huang P. J. Arif Y. Rempe M. P. Son J. J. John J. A. McDonald K. M. . (2025). High-definition transcranial direct-current stimulation of left primary motor cortices modulates beta and gamma oscillations serving motor control. J. Physiol. 603, 1627–1644. doi: 10.1113/JP287085, 40009440 PMC12177559

[ref76] Huang W. Chen Q. Liu L. Tang J. Zhou H. Tang Z. . (2023b). Clinical effect of short-term spinal cord stimulation in the treatment of patients with primary brainstem hemorrhage-induced disorders of consciousness. Front. Neurol. 14:1124871. doi: 10.3389/fneur.2023.1124871 37006496 PMC10064090

[ref77] Huang Z. Chen Y. Xiao Q. Kuang W. Liu K. Jiang Y. . (2022). Effect of acupuncture for disorders of consciousness in patients with stroke: a systematic review and meta-analysis. Front. Neurol. 13:930546. doi: 10.3389/fneur.2022.930546, 36277925 PMC9581330

[ref78] Huang Y. Xia X. Meng X. Bai Y. Feng Z. (2024). Single session of intermittent theta burst stimulation alters brain activity of patients in vegetative state. Aging 16, 7119–7130. doi: 10.18632/aging.205746, 38643463 PMC11087117

[ref79] Huang Y. Yang B. Liu X. Yang X. Gao J. Cui R. . (2025). Mechanisms and therapeutic potential of hyperbaric oxygen inducing autophagy in cerebral ischemia-reperfusion injury. Am. J. Transl. Res. 17, 9084–9091. doi: 10.62347/YXSV6853, 41415076 PMC12709312

[ref80] Huang W. Chen Q. Liu J. Liu L. Tang J. Zou M. . (2023a). Transcranial magnetic stimulation in disorders of consciousness: An update and perspectives. Aging Dis. 14, 1171–1183. doi: 10.14336/AD.2022.1114 37163434 PMC10389824

[ref81] Javid A. Ilham S. Kiani M. (2023). A review of ultrasound neuromodulation technologies. IEEE Trans Biomed Circuits Syst 17, 1084–1096. doi: 10.1109/TBCAS.2023.3299750 37506009

[ref82] Jia Y. He Y. F. Tian Y. Wang Y. Z. Zhao R. T. Li X. C. . (2022). MicroRNA alteration in cerebrospinal fluid from comatose patients with traumatic brain injury after right median nerve stimulation. Exp. Brain Res. 240, 2459–2470. doi: 10.1007/s00221-022-06414-7, 35933646

[ref83] Kazazian K. Monti M. M. Owen A. M. (2025). Functional neuroimaging in disorders of consciousness: towards clinical implementation. Brain 148, 2283–2298. doi: 10.1093/brain/awaf075, 39997570 PMC12233511

[ref84] Kondziella D. Bender A. Diserens K. van Erp W. Estraneo A. Formisano R. . (2020). European academy of neurology guideline on the diagnosis of coma and other disorders of consciousness. Eur. J. Neurol. 27, 741–756. doi: 10.1111/ene.14151 32090418

[ref85] Krugliakova E. Breuer F. Adelhöfer N. Alonso A. Besedovsky L. Murphy K. . (2025). Hacking the functions of sleep: non-invasive approaches to stimulate sleep neurophysiology. Physiol. Rev. 106, 675–749. doi: 10.1152/physrev.00007.2025 41263765

[ref86] Kuang N. Liu Z. Yu G. Wu X. Becker B. Fan H. . (2023). Neurodevelopmental risk and adaptation as a model for comorbidity among internalizing and externalizing disorders: genomics and cell-specific expression enriched morphometric study. BMC Med. 21:291. doi: 10.1186/s12916-023-02920-9, 37542243 PMC10403847

[ref87] Laureys S. (2005). The neural correlate of (un)awareness: lessons from the vegetative state. Trends Cogn. Sci. 9, 556–559. doi: 10.1016/j.tics.2005.10.010, 16271507

[ref88] Legon W. Ai L. Bansal P. Mueller J. K. (2018). Neuromodulation with single-element transcranial focused ultrasound in human thalamus. Hum. Brain Mapp. 39, 1995–2006. doi: 10.1002/hbm.23981 29380485 PMC6866487

[ref89] Legostaeva L. Poydasheva A. Iazeva E. Sinitsyn D. Sergeev D. Bakulin I. . (2019). Stimulation of the angular gyrus improves the level of consciousness. Brain Sci. 9:103. doi: 10.3390/brainsci9050103, 31064138 PMC6562708

[ref90] Li C. T. Cheng C. M. Lin H. C. Jeng J. S. Su T. P. Blumberger D. M. (2026). Prolonged intermittent theta burst stimulation as an alternative to standard rTMS: Real-world data from patients with non-psychotic major depression. Asian J. Psychiatr. 115:104794. doi: 10.1016/j.ajp.2025.104794, 41389597

[ref91] Li S. di Z. J. Liu Z. B. Zhao L. Li M. Y. Li H. L. (2024). Analysis of the efficacy of hyperbaric oxygen therapy for disorders of consciousness: a retrospective cohort study. Brain Behav. 14:e3588. doi: 10.1002/brb3.3588, 38945804 PMC11214873

[ref92] Li Y. Riganello F. Yu J. Vatrano M. Shen M. Cheng L. . (2025). The autonomic response following taVNS predicts changes in level of consciousness in DoC patients. Sci. Rep. 15:7317. doi: 10.1038/s41598-024-84029-4, 40025051 PMC11873156

[ref93] Li Y. Wan X. Zhang Y. Song W. (2024). Modulation of electroencephalogram brain activity dynamics by 10 Hz parietal repetitive transcranial magnetic stimulation: implications for recovery of the minimally conscious state. Neurosci. Lett. 842:137986. doi: 10.1016/j.neulet.2024.137986 39260738

[ref94] Lin Z. Cheng J. Tan C. Liu Z. Han D. (2025). Clinical development and guiding theory of transcranial magnetic stimulation: a literature review. Ann. Med. 57:2581921. doi: 10.1080/07853890.2025.2581921 41182327 PMC12584837

[ref95] Lissak I. A. Young M. J. (2024). Limitation of life sustaining therapy in disorders of consciousness: ethics and practice. Brain 147, 2274–2288. doi: 10.1093/brain/awae060, 38387081 PMC11224617

[ref96] Liu S. Gao Q. Guan M. Chen Y. Cheng S. Yang L. . (2022). Effectiveness of transcranial direct current stimulation over dorsolateral prefrontal cortex in patients with prolonged disorders of consciousness: a systematic review and meta-analysis. Front. Neurol. 13:998953. doi: 10.3389/fneur.2022.998953 36226076 PMC9549167

[ref97] Liu Y. S. Liu Z. B. Yang Z. Zhao L. Li H. L. (2022). Clinical efficacy of hyperbaric oxygen combined with different timings of right median-nerve electrical stimulation in patients with brain injury-induced disorders of consciousness. Brain Behav. 12:e2716. doi: 10.1002/brb3.2716, 35920129 PMC9480931

[ref98] Liu X. Meng F. Gao J. Zhang L. Zhou Z. Pan G. . (2018). Behavioral and resting state functional connectivity effects of high frequency rTMS on disorders of consciousness: a sham-controlled study. Front. Neurol. 9:982. doi: 10.3389/fneur.2018.00982 30519211 PMC6258881

[ref99] Liu S. Semyachkina-Glushkovskaya O. Yu T. Ilukov E. Rafailov E. Sokolovski S. . (2026). Neuro-lymphaphotonics opens new horizons of the future technologies for the therapy of brain diseases. Theranostics 16, 776–793. doi: 10.7150/thno.120374 41346709 PMC12674938

[ref100] Liu Z. Wu S. Wang S. Wu H. Gao H. Lu X. (2025). Can repetitive transcranial magnetic stimulation promote recovery of consciousness in patients with disorders of consciousness? A randomized controlled trial. Neuroimage Clin 46:103802. doi: 10.1016/j.nicl.2025.103802 40367603 PMC12142551

[ref101] Lucas R. J. Peirson S. N. Berson D. M. Brown T. M. Cooper H. M. Czeisler C. A. . (2014). Measuring and using light in the melanopsin age. Trends Neurosci. 37, 1–9. doi: 10.1016/j.tins.2013.10.004, 24287308 PMC4699304

[ref102] Luo J. Tan Z. Shang P. Huang S. Liu Y. Wang Y. . (2025). Accelerated intermittent theta burst stimulation combined with cognitive training modulates cortical plasticity and brain activation in patients with amnestic mild cognitive impairment. Exp. Gerontol. 213:113009. doi: 10.1016/j.exger.2025.113009 41423172

[ref103] Ma X. Qi Y. Xu C. Weng Y. Yu J. Sun X. . (2024). How well do neural signatures of resting-state EEG detect consciousness? A large-scale clinical study. Hum. Brain Mapp. 45:e26586. doi: 10.1002/hbm.26586 38433651 PMC10910334

[ref104] Ma L. Wang H. B. Hashimoto K. (2025). The vagus nerve: An old but new player in brain-body communication. Brain Behav. Immun. 124, 28–39. doi: 10.1016/j.bbi.2024.11.023 39566667

[ref105] Ma W. Wang F. Yi Y. Huang Y. Li X. Liu Y.'. . (2024). Mapping the electric field of high-definition transcranial electrical stimulation across the lifespan. Sci. Bull. 69, 3876–3888. doi: 10.1016/j.scib.2024.10.001, 39424454

[ref106] Ma Q. Liu C. Zhao G. Guo S. Li H. Zhang B. . (2025). Acupuncture for insomnia in people with cancer. Cochrane Database Syst. Rev. 2025:Cd015177. doi: 10.1002/14651858.CD015177.pub2 PMC12679689 41347621

[ref107] Magliacano A. de Bellis F. Panico F. Sagliano L. Trojano L. Sandroni C. . (2023). Long-term clinical evolution of patients with prolonged disorders of consciousness due to severe anoxic brain injury: a meta-analytic study. Eur. J. Neurol. 30, 3913–3927. doi: 10.1111/ene.15899, 37246500

[ref108] Majdi A. Chen L. Larsen L. E. Raedt R. Laughlin M. M. (2025). tDCS cranial nerve co-stimulation: unveiling brainstem pathways involved in trigeminal nerve direct current stimulation in rats. Brain Stimul. 18, 171–184. doi: 10.1016/j.brs.2025.01.025, 39921050 PMC12012264

[ref109] Mancuso M. Abbruzzese L. Canova S. Landi G. Rossi S. Santarnecchi E. (2017). Transcranial random noise stimulation does not improve behavioral and neurophysiological measures in patients with subacute vegetative-unresponsive wakefulness state (VS-UWS). Front. Hum. Neurosci. 11:524. doi: 10.3389/fnhum.2017.00524, 29163104 PMC5681535

[ref110] Martens G. Kroupi E. Bodien Y. Frasso G. Annen J. Cassol H. . (2020). Behavioral and electrophysiological effects of network-based frontoparietal tDCS in patients with severe brain injury: a randomized controlled trial. Neuroimage Clin 28:102426. doi: 10.1016/j.nicl.2020.102426, 32977212 PMC7511767

[ref111] Martens G. Lejeune N. O'Brien A. T. Fregni F. Martial C. Wannez S. . (2018). Randomized controlled trial of home-based 4-week tDCS in chronic minimally conscious state. Brain Stimul. 11, 982–990. doi: 10.1016/j.brs.2018.04.021 29759943

[ref112] Mashour G. A. Pal D. Brown E. N. (2022). Prefrontal cortex as a key node in arousal circuitry. Trends Neurosci. 45, 722–732. doi: 10.1016/j.tins.2022.07.002, 35995629 PMC9492635

[ref113] Merzenich M. M. Jenkins W. M. (1993). Reorganization of cortical representations of the hand following alterations of skin inputs induced by nerve injury, skin island transfers, and experience. J. Hand Ther. 6, 89–104. doi: 10.1016/S0894-1130(12)80290-0, 8393727

[ref114] Mitterová K. Pupíková M. Gajdoš M. Eliášová I. Rektorová I. (2025). Optimizing tACS for Working memory: differential Outcomes in Healthy Aging and non-amnestic mild Cognitive Impairment. Alzheimers Res Ther 18:1922. doi: 10.1186/s13195-025-01922-4 PMC12777262 41331881

[ref115] Montazeri K. Farhadi M. Fekrazad R. Akbarnejad Z. Chaibakhsh S. Mahmoudian S. (2021). Transcranial photobiomodulation in the management of brain disorders. J. Photochem. Photobiol. B 221:112207. doi: 10.1016/j.jphotobiol.2021.112207, 34119804

[ref116] Monti M. M. Rosenberg M. Finoia P. Kamau E. Pickard J. D. Owen A. M. (2015). Thalamo-frontal connectivity mediates top-down cognitive functions in disorders of consciousness. Neurology 84, 167–173. doi: 10.1212/WNL.0000000000001123, 25480912 PMC4336082

[ref117] Monti M. M. Schnakers C. Korb A. S. Bystritsky A. Vespa P. M. (2016). Non-invasive ultrasonic thalamic stimulation in disorders of consciousness after severe brain injury: a first-in-man report. Brain Stimul. 9, 940–941. doi: 10.1016/j.brs.2016.07.008 27567470

[ref118] Motsenyat A. Zhong X. Z. van Lankveld H. Chen J. X. Mathew A. Chen J. J. (2025). Modulating cerebrospinal fluid dynamics using pulsed photobiomodulation. Brain Stimul. 19:102988. doi: 10.1016/j.brs.2025.102988 41314512

[ref119] Mullinger K. J. Mayhew S. D. Bagshaw A. P. Bowtell R. Francis S. T. (2013). Poststimulus undershoots in cerebral blood flow and BOLD fMRI responses are modulated by poststimulus neuronal activity. Proc. Natl. Acad. Sci. USA 110, 13636–13641. doi: 10.1073/pnas.1221287110, 23898206 PMC3746922

[ref120] Naeser M. A. Saltmarche A. Krengel M. H. Hamblin M. R. Knight J. A. (2011). Improved cognitive function after transcranial, light-emitting diode treatments in chronic, traumatic brain injury: two case reports. Photomed. Laser Surg. 29, 351–358. doi: 10.1089/pho.2010.2814 21182447 PMC3104287

[ref121] Naro A. Bramanti P. Leo A. Russo M. Calabrò R. S. (2016). Transcranial alternating current stimulation in patients with chronic disorder of consciousness: a possible way to cut the diagnostic Gordian knot? Brain Topogr. 29, 623–644. doi: 10.1007/s10548-016-0489-z, 27062669

[ref122] Nejati V. Vaziri Z. Antal A. Antonenko D. Behroozmand R. Bestmann S. . (2025). Report approval for transcranial electrical stimulation (RATES): expert recommendation based on a Delphi consensus study. Nat. Protoc. doi: 10.1038/s41596-025-01259-0 41044265

[ref123] Noé E. Ferri J. Colomer C. Moliner B. O'Valle M. Ugart P. . (2020). Feasibility, safety and efficacy of transauricular vagus nerve stimulation in a cohort of patients with disorders of consciousness. Brain Stimul. 13, 427–429. doi: 10.1016/j.brs.2019.12.005, 31866491

[ref124] Olaitan G. Ganesana M. Strohman A. Lynch W. J. Legon W. Venton B. J. (2025). Focused ultrasound modulates dopamine in a mesolimbic reward circuit. J. Neurochem. 169:e70001. doi: 10.1111/jnc.70001, 39902479 PMC11791541

[ref125] Osińska A. Rynkiewicz A. Binder M. Komendziński T. Borowicz A. Leszczyński A. (2022). Non-invasive Vagus nerve stimulation in treatment of disorders of consciousness - longitudinal case study. Front. Neurosci. 16:834507. doi: 10.3389/fnins.2022.834507, 35600632 PMC9120963

[ref126] Othman M. H. Toury-Puel A. G. Hansen K. I. T. Amiri M. Zarifkar P. Peinkhofer C. . (2025). Stimulants for disorders of consciousness in the intensive care unit: a randomized, placebo-controlled trial. Brain 148, 3523–3536. doi: 10.1093/brain/awaf228, 40501148 PMC12493042

[ref127] Owens M. M. Jacquemet V. Napadow V. Lewis N. Beaumont E. (2024). Brainstem neuronal responses to transcutaneous auricular and cervical vagus nerve stimulation in rats. J. Physiol. 602, 4027–4052. doi: 10.1113/JP286680, 39031516 PMC11326965

[ref128] Peri C. V. Shaffrey M. E. Farace E. Cooper E. Alves W. M. Cooper J. B. . (2001). Pilot study of electrical stimulation on median nerve in comatose severe brain injured patients: 3-month outcome. Brain Inj. 15, 903–910. doi: 10.1080/02699050110065709, 11595086

[ref129] Piedade G. S. Assumpcao de Monaco B. Guest J. D. Cordeiro J. G. (2023). Review of spinal cord stimulation for disorders of consciousness. Curr. Opin. Neurol. 36, 507–515. doi: 10.1097/WCO.0000000000001222, 37889524

[ref130] Ping A. A. Guan L. Z. Wang Y. Yang S. Yang C. Hu X. Q. . (2025). A methodological guideline for consciousness assessment via neural electrophysiological activity. Mil. Med. Res. 12:90. doi: 10.1186/s40779-025-00682-4 41382202 PMC12699880

[ref131] Reale G. Fusco A. Cocciolillo F. Amoruso V. Glorioso D. Caputo M. . (2025). Live effects of anodal and cathodal transcranial direct current stimulation on brain metabolism in a patient with typical hemorrhagic stroke: a case study. Brain Sci. 15:594. doi: 10.3390/brainsci15060594, 40563766 PMC12191415

[ref132] Rosanova M. Fecchio M. Casarotto S. Sarasso S. Casali A. G. Pigorini A. . (2018). Sleep-like cortical OFF-periods disrupt causality and complexity in the brain of unresponsive wakefulness syndrome patients. Nat. Commun. 9:4427. doi: 10.1038/s41467-018-06871-1, 30356042 PMC6200777

[ref133] Rubin D. B. (2025). A circuit level investigation into the neuromodulatory effect of spinal cord stimulation for motor recovery. Neuron 113, 3695–3696. doi: 10.1016/j.neuron.2025.10.013, 41265396

[ref134] Schiff N. D. (2010). Recovery of consciousness after brain injury: a mesocircuit hypothesis. Trends Neurosci. 33, 1–9. doi: 10.1016/j.tins.2009.11.002, 19954851 PMC2931585

[ref135] Schiff N. D. (2023). Mesocircuit mechanisms in the diagnosis and treatment of disorders of consciousness. Presse Med. 52:104161. doi: 10.1016/j.lpm.2022.104161, 36563999

[ref136] Schuler A. L. Tik M. Kallioniemi E. Suller Marti A. Cai Z. Pellegrino G. (2025). Approaches to map cortical excitability beyond the primary motor cortex - perspectives from cognitive neuroscience, multimodal imaging and clinical applications. Neurosci. Biobehav. Rev. 177:106338. doi: 10.1016/j.neubiorev.2025.106338, 40812727

[ref137] Shen L. Huang Y. Liao Y. Yin X. Huang Y. Ou J. . (2023). Effect of high-frequency repetitive transcranial magnetic stimulation over M1 for consciousness recovery after traumatic brain injury. Brain Behav. 13:e2971. doi: 10.1002/brb3.2971, 36977194 PMC10176007

[ref138] Shi Y. Cai G. Wu W. (2025). A panoramic review of transcranial focused ultrasound neuromodulation: from basic research to clinical applications. J. Neuroeng. Rehabil. 22:227. doi: 10.1186/s12984-025-01753-2, 41152970 PMC12570825

[ref139] Shi A. Li W. Liu X. Lu J. Guo J. (2025). Acupuncture for depression: decoding neuroimmune crosstalk and targeting anti-inflammatory mechanisms. Curr. Opin. Pharmacol. 86:102585. doi: 10.1016/j.coph.2025.102585 41349277

[ref140] Sierra A. Barra A. Navarro M. D. Ferri J. Noé E. Llorens R. (2025). Long-term Progress of functional Independence of patients with prolonged disorders of consciousness who emerge from the minimally conscious state. Arch. Phys. Med. Rehabil. doi: 10.1016/j.apmr.2025.11.012 41309024

[ref141] Song J. Torres-Carmona E. Abdolizadeh A. Kambari Y. Amaev A. Ueno F. . (2025). The effect of single-session transcranial direct current stimulation on cerebral blood flow: a systematic review and Meta-analysis. Brain Topogr. 38:60. doi: 10.1007/s10548-025-01140-z, 40875070

[ref142] Sorum B. Rietmeijer R. A. Gopakumar K. Adesnik H. Brohawn S. G. (2021). Ultrasound activates mechanosensitive TRAAK K(+) channels through the lipid membrane. Proc. Natl. Acad. Sci. USA 118:118. doi: 10.1073/pnas.2006980118 PMC8017979 33542098

[ref143] Spaccavento S. Carraturo G. Brattico E. Matarrelli B. Rivolta D. Montenegro F. . (2024). Musical and electrical stimulation as intervention in disorder of consciousness (DOC) patients: a randomised cross-over trial. PLoS One 19:e0304642. doi: 10.1371/journal.pone.0304642, 38820520 PMC11142721

[ref144] Straudi S. Antonioni A. Baroni A. Bonsangue V. Lavezzi S. Koch G. . (2023). Anti-inflammatory and cortical responses after transcranial direct current stimulation in disorders of consciousness: An exploratory study. J. Clin. Med. 13:108. doi: 10.3390/jcm13010108, 38202115 PMC10779892

[ref145] Sun F. Liu W. Li X. Wang X. Ou Y. Li X. . (2024). Median nerve electrical stimulation improves traumatic brain injury by reducing TACR1 to inhibit nuclear factor-κB and CCL7 activation in microglia. Histol. Histopathol. 39, 889–902. doi: 10.14670/HH-18-686, 38098319

[ref146] Sun J. Yan J. Zhao L. Wei X. Qiu C. Dong W. . (2024). Spinal cord stimulation for prolonged disorders of consciousness: a study on scalp electroencephalography. CNS Neurosci. Ther. 30:e70180. doi: 10.1111/cns.70180 39736021 PMC11683476

[ref147] Sveva V. Mancuso M. Cruciani A. Casula E. P. Leodori G. Selvaggi S. A. . (2025). Cellular and molecular mechanisms of non-invasive brain stimulation techniques: a systematic review on the implications for the treatment of neurological disorders. Cells 14:1996. doi: 10.3390/cells14241996, 41440017 PMC12732073

[ref148] Tang Q. He M. Zheng P. Sun M. Cao J. Zhang Q. . (2025). Brain TRPV1 channel-mediated calcium influx: the immunomodulatory pathway of acupuncture in neuroinflammation. Front. Immunol. 16:1700282. doi: 10.3389/fimmu.2025.1700282, 41459513 PMC12738356

[ref149] Teasdale G. Jennett B. (1974). Assessment of coma and impaired consciousness. A practical scale. Lancet 2, 81–84. doi: 10.1016/S0140-6736(74)91639-0, 4136544

[ref150] Tedford C. E. DeLapp S. Jacques S. Anders J. (2015). Quantitative analysis of transcranial and intraparenchymal light penetration in human cadaver brain tissue. Lasers Surg. Med. 47, 312–322. doi: 10.1002/lsm.22343 25772014

[ref151] Thibaut A. Bodien Y. G. Laureys S. Giacino J. T. (2020). Minimally conscious state "plus": diagnostic criteria and relation to functional recovery. J. Neurol. 267, 1245–1254. doi: 10.1007/s00415-019-09628-y, 31773246

[ref152] Thibaut A. Chennu S. Chatelle C. Martens G. Annen J. Cassol H. . (2018). Theta network centrality correlates with tDCS response in disorders of consciousness. Brain Stimul. 11, 1407–1409. doi: 10.1016/j.brs.2018.09.002, 30241773

[ref153] Thibaut A. Fregni F. Estraneo A. Fiorenza S. Noe E. Llorens R. . (2023). Sham-controlled randomized multicentre trial of transcranial direct current stimulation for prolonged disorders of consciousness. Eur. J. Neurol. 30, 3016–3031. doi: 10.1111/ene.15974 37515394

[ref154] Tittelmeier J. Kaub L. Milz S. Kugelmann D. Hof P. R. Schmitz C. . (2025). Insufficient low-level near infrared light penetration challenges the efficacy of transcranial photobiomodulation. Brain Stimul. 18, 1220–1223. doi: 10.1016/j.brs.2025.07.001, 40614861

[ref155] Tong C. Li W. Zou Y. Xia Y. Pei M. Zhang K. . (2025). Norepinephrine-mediated arousal fluctuations drive inverted U-shaped functional connectivity dynamics. Nat. Commun. 16:11318. doi: 10.1038/s41467-025-66436-x, 41390822 PMC12722237

[ref156] Tsai Y. J. Lin C. T. Lue J. H. (2007). Characterization of the induced neuropeptide Y-like immunoreactivity in primary sensory neurons following complete median nerve transection. J. Neurotrauma 24, 1878–1888. doi: 10.1089/neu.2007.3488, 18159999

[ref157] Uner S. Akdogan I. Pavan A. Kafaligonul H. (2025). The hidden benefits of noise: low-frequency tRNS and dynamic visual noise enhance visual processing. J. Neurosci. 45:e0853252025. doi: 10.1523/JNEUROSCI.0853-25.2025, 41167815 PMC12660171

[ref158] Vitello M. M. Briand M. M. Ledoux D. Annen J. el Tahry R. Laureys S. . (2023). Transcutaneous vagal nerve stimulation to treat disorders of consciousness: protocol for a double-blind randomized controlled trial. Int. J. Clin. Health Psychol. 23:100360. doi: 10.1016/j.ijchp.2022.100360, 36467262 PMC9712558

[ref159] Vitello M. M. Rosenfelder M. J. Cardone P. Niimi M. Willacker L. Thibaut A. . (2023). A protocol for a multicenter randomized and personalized controlled trial using rTMS in patients with disorders of consciousness. Front. Neurol. 14:1216468. doi: 10.3389/fneur.2023.1216468 37545735 PMC10401598

[ref160] Wan X. Zhang Y. Li Y. Song W. (2024a). Effects of parietal repetitive transcranial magnetic stimulation in prolonged disorders of consciousness: a pilot study. Heliyon 10:e30192. doi: 10.1016/j.heliyon.2024.e30192, 38707352 PMC11066627

[ref161] Wan X. Zhang Y. Li Y. Song W. (2024b). An update on noninvasive neuromodulation in the treatment of patients with prolonged disorders of consciousness. CNS Neurosci. Ther. 30:e14757. doi: 10.1111/cns.14757, 38747078 PMC11094579

[ref162] Wang P. Cao W. Zhou H. Zhang H. X. Zhang L. Liu L. . (2022). Efficacy of median nerve electrical stimulation on the recovery of patients with consciousness disorders: a systematic review and meta-analysis. J. Int. Med. Res. 50:3000605221134467. doi: 10.1177/03000605221134467, 36448965 PMC9720824

[ref163] Wang Y. Dang Y. Bai Y. Xia X. Li X. (2023). Evaluating the effect of spinal cord stimulation on patient with disorders of consciousness: a TMS-EEG study. Comput. Biol. Med. 166:107547. doi: 10.1016/j.compbiomed.2023.107547 37806053

[ref164] Wang X. Guo Y. Zhang Y. Li J. Gao Z. Li Y. . (2020). Combined behavioral and mismatch negativity evidence for the effects of Long-lasting high-definition tDCS in disorders of consciousness: a pilot study. Front. Neurosci. 14:381. doi: 10.3389/fnins.2020.00381, 32410950 PMC7198816

[ref165] Wang J. Lai Q. Han J. Qin P. Wu H. (2024). Neuroimaging biomarkers for the diagnosis and prognosis of patients with disorders of consciousness. Brain Res. 1843:149133. doi: 10.1016/j.brainres.2024.149133, 39084451

[ref166] Wang H. Y. Tsai Y. J. Chen S. H. Lin C. T. Lue J. H. (2012). Nitric oxide implicates c-Fos expression in the cuneate nucleus following electrical stimulation of the transected median nerve. Neurochem. Res. 37, 84–95. doi: 10.1007/s11064-011-0585-0, 21892689

[ref167] Wei J. Zou H. Tang Q. Yao Z. Huang G. Liang Z. . (2025). Enhancing visual perception by modulating prestimulus alpha and beta power with tRNS. Commun Biol 8:1182. doi: 10.1038/s42003-025-08600-z, 40781150 PMC12334615

[ref168] Williams A. Bass G. D. Hampton S. Klinedinst R. Giacino J. T. Fischer D. (2025). Delayed withdrawal of life-sustaining treatment in disorders of consciousness: practical and theoretical considerations. Neurocrit. Care. 42, 1064–1073. doi: 10.1007/s12028-024-02143-7, 39407075 PMC12137379

[ref169] Wislowska M. del Giudice R. Lechinger J. Wielek T. Heib D. P. J. Pitiot A. . (2017). Night and day variations of sleep in patients with disorders of consciousness. Sci. Rep. 7:266. doi: 10.1038/s41598-017-00323-4, 28325926 PMC5428269

[ref170] Wu X. Xie L. Lei J. Yao J. Li J. Ruan L. . (2023). Acute traumatic coma awakening by right median nerve electrical stimulation: a randomised controlled trial. Intensive Care Med. 49, 633–644. doi: 10.1007/s00134-023-07072-1 37178149 PMC10182548

[ref171] Wu X. Zhang K. Kuang N. Kong X. Cao M. Lian Z. . (2025). Developing brain asymmetry shapes cognitive and psychiatric outcomes in adolescence. Nat. Commun. 16:4480. doi: 10.1038/s41467-025-59110-9, 40368909 PMC12534418

[ref172] Wu Y. Zhao K. Wen W. Zhu K. Lu F.’. Kong Y. . (2024). Acupuncture for poststroke coma: a systematic review and meta-analysis. Complement. Ther. Med. 82:103046. doi: 10.1016/j.ctim.2024.103046 38704101

[ref173] Xia X. Bai Y. Zhou Y. Yang Y. Xu R. Gao X. . (2017). Effects of 10 Hz repetitive transcranial magnetic stimulation of the left dorsolateral prefrontal cortex in disorders of consciousness. Front. Neurol. 8:182. doi: 10.3389/fneur.2017.00182, 28515709 PMC5413493

[ref174] Xiong Q. le K. Tang Y. Ye W. Wang Y. Zhong Y. . (2023). Effect of single and combined median nerve stimulation and repetitive transcranial magnetic stimulation in patients with prolonged disorders of consciousness: a prospective, randomized, single-blinded, controlled trial. Front. Aging Neurosci. 15:1112768. doi: 10.3389/fnagi.2023.1112768, 37168716 PMC10164991

[ref175] Xu C. Wu W. Zheng X. Liang Q. Huang X. Zhong H. . (2023). Repetitive transcranial magnetic stimulation over the posterior parietal cortex improves functional recovery in nonresponsive patients: a crossover, randomized, double-blind, sham-controlled study. Front. Neurol. 14:1059789. doi: 10.3389/fneur.2023.1059789, 36873436 PMC9978157

[ref176] Xu C. Yuan Z. Chen Z. Liao Z. Li S. Feng Y. . (2024). Perturbational complexity index in assessing responsiveness to rTMS treatment in patients with disorders of consciousness: a cross-over randomized controlled trial study. J. Neuroeng. Rehabil. 21:167. doi: 10.1186/s12984-024-01455-1, 39300529 PMC11411826

[ref177] Xun X. Liu Y. Pan W. Tang L. Hu C. Ouyang H. . (2025). Low frequency-repetitive transcranial magnetic stimulation combined with Xingnao Kaiqiao acupuncture improves post-stroke cognitive impairment and has better clinical efficacy. Psychogeriatrics 25:e13199. doi: 10.1111/psyg.13199 39462185

[ref178] Yang D. Fu S. Zhao M. Shi Y. (2025). The promise of transcranial focused ultrasound in disorders of consciousness: a narrative review. Crit. Care 29:109. doi: 10.1186/s13054-025-05338-2, 40075493 PMC11905659

[ref179] Yang Y. He Q. Xia X. Dang Y. Chen X. He J. . (2022). Long-term functional prognosis and related factors of spinal cord stimulation in patients with disorders of consciousness. CNS Neurosci. Ther. 28, 1249–1258. doi: 10.1111/cns.13870 35619213 PMC9253730

[ref180] Yang J. Li X. Yang X. Zhu T. Ou S. (2025). Acute traumatic coma awakening induced by median nerve electrical stimulation: a systematic review and Meta-analysis. Neurocrit. Care. 42, 817–828. doi: 10.1007/s12028-024-02141-9, 39448428

[ref181] Yang Y. Luo Y. Feng M. Luo P. Zeng J. Shi X. . (2024). Median nerve electrical stimulation for restoring consciousness in patients with traumatic brain injury: study protocol for a systematic review and meta-analysis. BMJ Open 14:e091560. doi: 10.1136/bmjopen-2024-091560, 39532381 PMC11574438

[ref182] Yang L. Zhu H. Z. Xie L. Yang J. Wei H. T. Yu Q. T. . (2025). Neuroprotective effects of hyperbaric oxygen therapy on vascular cognitive impairment in hypoperfused mice via miR-137-3p/TRAF3 pathway. Transl. Psychiatry 15:537. doi: 10.1038/s41398-025-03771-z, 41318710 PMC12727875

[ref183] Yelden K. James L. M. Duport S. Kempny A. Farmer S. F. Leff A. P. . (2022). A simple intervention for disorders of consciousness- is there a light at the end of the tunnel? Front. Neurol. 13:824880. doi: 10.3389/fneur.2022.824880, 35937075 PMC9355643

[ref184] Yifei W. Yi Y. Yu W. Jinling Z. Weihang Z. Shaoyuan L. . (2022). Transcutaneous auricular vague nerve stimulation improved brain connection activity on patients of disorders of consciousness: a pilot study. J. Tradit. Chin. Med. 42, 463–471. doi: 10.19852/j.cnki.jtcm.2022.03.012 35610018 PMC9924658

[ref185] Yoo S. Mittelstein D. R. Hurt R. C. Lacroix J. Shapiro M. G. (2022). Focused ultrasound excites cortical neurons via mechanosensitive calcium accumulation and ion channel amplification. Nat. Commun. 13:493. doi: 10.1038/s41467-022-28040-1, 35078979 PMC8789820

[ref186] Yu G. Liu Z. Wu X. Becker B. Zhang K. Fan H. . (2023). Common and disorder-specific cortical thickness alterations in internalizing, externalizing and thought disorders during early adolescence: an adolescent brain and cognitive development study. J. Psychiatry Neurosci. 48, E345–e356. doi: 10.1503/jpn.220202, 37673436 PMC10495167

[ref187] Yu Y. Yang Y. Gan S. Guo S. Fang J. Wang S. . (2021). Cerebral hemodynamic correlates of transcutaneous auricular vagal nerve stimulation in consciousness restoration: An open-label pilot study. Front. Neurol. 12:684791. doi: 10.3389/fneur.2021.684791 34335449 PMC8319239

[ref188] Yu Y. T. Yang Y. Wang L. B. Fang J. L. Chen Y. Y. He J. H. . (2017). Transcutaneous auricular vagus nerve stimulation in disorders of consciousness monitored by fMRI: the first case report. Brain Stimul. 10, 328–330. doi: 10.1016/j.brs.2016.12.004, 28017322

[ref189] Zhang X. H. Han P. Zeng Y. Y. Wang Y. L. Lv H. L. (2021). The clinical effect of repetitive transcranial magnetic stimulation on the disturbance of consciousness in patients in a vegetative state. Front. Neurosci. 15:647517. doi: 10.3389/fnins.2021.647517, 33994925 PMC8119637

[ref190] Zhang X. Liu B. Li Y. Duan G. Hou J. Wu D. (2021). Multi-target and multi-session transcranial direct current stimulation in patients with prolonged disorders of consciousness: a controlled study. Front. Neurosci. 15:641951. doi: 10.3389/fnins.2021.641951, 34566555 PMC8456025

[ref191] Zhang X. Miao X. Jiang H. Ren Y. Huo L. Liu M. . (2025). Advanced intervention effects of pulsed and steady transcranial Photobiomodulation on sleep, mood, and EEG signal regulation. J. Biophotonics 18:e70004. doi: 10.1002/jbio.70004, 40101768

[ref192] Zhang Y. Wan X. Wang Y. Li X. du J. Yan X. . (2025). Parietal rTMS induced changes in cortical excitability in patients with minimally conscious state using TMS-EEG. CNS Neurosci. Ther. 31:e70583. doi: 10.1111/cns.70583 40904190 PMC12409070

[ref193] Zhao L. Li S. Liu Y. di Z. Li H. (2025). Analysis of prognostic risk factors in children with disorders of consciousness undergoing hyperbaric oxygen therapy. J. Multidiscip. Healthc. 18, 3803–3812. doi: 10.2147/JMDH.S517708, 40621186 PMC12227008

[ref194] Zhao Z. Wang Y. Xia X. Li X. (2024). Permutation conditional mutual information to quantify TMS-evoked cortical connectivity in disorders of consciousness. J. Neural Eng. 21:046029. doi: 10.1088/1741-2552/ad618b, 38986463

[ref195] Zhou Y. Sun Y. He P. Xiong Q. Kang J. Tang Y. . (2023). The efficacy and safety of transcutaneous auricular vagus nerve stimulation for patients with minimally conscious state: a sham-controlled randomized double-blind clinical trial. Front. Neurosci. 17:1323079. doi: 10.3389/fnins.2023.1323079, 38156271 PMC10752952

[ref196] Zhuang Y. Ge Q. Li Q. Xu L. Geng X. Wang R. . (2024). Combined behavioral and EEG evidence for the 70 Hz frequency selection of short-term spinal cord stimulation in disorders of consciousness. CNS Neurosci. Ther. 30:e14388. doi: 10.1111/cns.14388, 37563991 PMC10848050

[ref197] Zhuang Y. Yang Y. Xu L. Chen X. Geng X. Zhao J. . (2022). Effects of short-term spinal cord stimulation on patients with prolonged disorder of consciousness: a pilot study. Front. Neurol. 13:1026221. doi: 10.3389/fneur.2022.1026221, 36313512 PMC9614028

[ref198] Zhuang Y. Zhai W. Li Q. Jiao H. Ge Q. Rong P. . (2023). Effects of simultaneous transcutaneous auricular vagus nerve stimulation and high-definition transcranial direct current stimulation on disorders of consciousness: a study protocol. Front. Neurol. 14:1165145. doi: 10.3389/fneur.2023.1165145, 37693756 PMC10483839

[ref200] Zou N. Zhou Q. Zhang Y. Xin C. Wang Y. Claire-Marie R. . (2024). Transcutaneous auricular vagus nerve stimulation as a novel therapy connecting the central and peripheral systems: a review. Int. J. Surg. 110, 4993–5006. doi: 10.1097/JS9.0000000000001592, 38729100 PMC11326027

